# Integrating intestinal microbiome and urinary metabolome data to predict secondary infection in critically ill patients

**DOI:** 10.1186/s13054-025-05818-5

**Published:** 2026-03-13

**Authors:** Charlotte Linz, Kristiyana Tsenova, Katja Dettmer, Lisa Ellmann, Peter J. Oefner, Wolfram Gronwald, Fedja Farowski, Alina M. Rüb, Daniel E. Freedberg, Philipp Koehler, Jorge Garcia Borrega, Jan-Hendrik Naendrup, Maria J. G. T. Vehreschild, Boris Böll

**Affiliations:** 1 Division of Hematology-Oncology/Critical Care Medicine/Infectious Diseases, Faculty of Medicine and University Hospital Cologne, Department I of Internal Medicine, Center for Integrated Oncology Aachen Bonn Cologne Düsseldorf (CIO ABCD), University of Cologne, Kerpener Strasse 62, Cologne, Germany; 2 Department II of Internal Medicine, Infectious Diseases, University Hospital Frankfurt, Goethe University Frankfurt, Frankfurt am Main, Germany; 3 Institute of Functional Genomics, University of Regensburg, Regensburg, Germany; 4 Division of Digestive and Liver Diseases, Mailman School of Public Health, Department of Epidemiology, Columbia University, New York, USA; 5 Division of Clinical Immunology, Faculty of Medicine and University Hospital Cologne, Department I of Internal Medicine, University of Cologne, Cologne, Germany; 6 Faculty of Medicine and University Hospital Cologne, Institute of Translational Research, Cologne Excellence Cluster on Cellular Stress Responses in Aging-Associated Diseases (CECAD), University of Cologne, Cologne, Germany

**Keywords:** Secondary infection, Ventilator-associated pneumonia (VAP), Bloodstream infection (BSI), Intestinal microbiome, Urinary metabolites

## Abstract

**Background:**

Secondary infection (SI), including ventilator-associated pneumonia (VAP) and bloodstream infection (BSI), represents a major complication in critically ill patients. Current clinical risk stratification approaches prove inadequate for timely and precise identification of at-risk patients. This study identifies intestinal microbiome and urinary metabolome characteristics (“multi-omics data”) associated with SI occurrence, investigates convergence of the respiratory microbiome with the intestinal microbiome, and determines whether multi-omics integration enhances prognostic discrimination for patients at risk of developing SI.

**Methods:**

We analyzed data from mechanically ventilated patients from two cohorts: University Hospital Cologne (UHC), Germany, and Columbia University Medical Center (CUMC), New York, United States. The core dataset (*n* = 88; 64 UHC and 24 CUMC) assessed multi-omics integration for SI prediction, with an UHC subset (*n* = 55) providing more comprehensive clinical and microbiome characterization. Baseline intestinal and respiratory microbiome, as well as urinary metabolome data were collected within 48 h of intensive care unit admission or intubation using 16 S ribosomal ribonucleic acid (rRNA) sequencing and nuclear magnetic resonance (NMR) spectroscopy. SI was defined as new-onset BSI or VAP occurring ≥ 48 h after enrollment. Regression and classification models compared clinical-only approaches with integrated multi-omics models using model selection criteria, area under the curve (AUC), and Matthews correlation coefficients.

**Results:**

SI occurred in 28% of patients, with prior antibiotic exposure associated with SI (84% vs. 41%, *q* < 0.01; odds ratio 2.57, *p* = 0.17). SI patients exhibited significantly lower baseline intestinal microbial diversity (Shannon diversity, 1.96 vs. 3.47, *p* < 0.01) and greater *Enterococcus* abundance (46% vs. 11%, *q* = 0.02), with similar patterns observed in the respiratory microbiome. Urinary NMR analysis identified metabolites mapping to features at 0.935 ppm (2-oxoisocaproate, isoleucine) in the core dataset, and at 8.025 ppm (quinolinate) in the UHC subset as elevated in SI patients. Multi-omics models demonstrated modest but consistent improvement over clinical-only models (AUC: 0.75 vs. 0.64).

**Conclusions:**

SI susceptibility in critically ill patients associates with underlying clinical severity, prior antibiotic exposure, and microbiota disruption. Multi-omics integration yielded consistent predictive improvement, supporting prospective validation as a proof-of-concept approach for early SI risk stratification.

**Supplementary Information:**

The online version contains supplementary material available at 10.1186/s13054-025-05818-5.

## Background

Secondary infection (SI), notably ventilator-associated pneumonia (VAP) and bloodstream infection (BSI), significantly increases morbidity, mortality, and healthcare costs in critically ill patients [1, 2]. Over half of intensive care unit (ICU) patients experience at least one infection during their stay, with approximately one-fifth classified as ICU-acquired. Associated mortality rates reach 28‒34% in hospitalized populations [1]. The emergence of multidrug-resistant pathogens further constrains therapeutic options, emphasizing the need for enhanced early risk identification and preventive strategies [3].

The intestinal microbiota plays a central role in modulating host susceptibility to SI through mechanisms of colonization resistance. Many nosocomial pathogens originate from this reservoir; however, not all colonized patients develop active infections. This protective function relies on commensal anaerobes that restrict pathogen expansion via nutrient competition, antimicrobial compound production, and bacteriophage activity [4, 5]. Fecal biodiversity is often considered a surrogate measure of this resistance. Reduced microbial diversity and pathogen overgrowth have been associated with increased infection risk, disease severity, and mortality in ICU populations [6–9]. Antibiotic exposure, a nearly universal feature in critical illness, further disrupts these protective communities and favors pathogen proliferation [6].

Despite these well-established associations, baseline microbiome features have not been systematically evaluated for their independent predictive value in SI development. This uncertainty reflects methodological variability, limited longitudinal data establishing causality, and inter-individual heterogeneity in dysbiotic responses. The predictive value of baseline microbiome features for infection remains unclear [10, 11]. Additionally, current sequencing technologies remain unsuitable for real-time clinical deployment. Consequently, non-invasive urinary metabolites warrant investigation as potential surrogate markers of microbiota integrity. Previous work has identified associations between depleted urinary 3-indoxyl sulfate (3-IS), a tryptophan-derived metabolite produced by intestinal commensal bacteria, and adverse outcomes, including gut microbiota disruption and increased one-year mortality, both following allogeneic stem cell transplantation [12] and ICU admission for mainly sepsis, post-surgery or cardiac causes [13].

Therefore, this study addresses three interconnected objectives: [1] to identify intestinal microbiome and urinary metabolome signatures (“multi-omics data”) associated with SI development [2], to investigate the convergence of the respiratory microbiome with the intestinal microbiome, and [3] to evaluate whether integrating multi-omics data with clinical variables enhances predictive discrimination compared to clinical assessment alone.

## Patients and methods

### Study framework and patient cohorts

This analysis draws on data from two independent tertiary-care academic cohorts: the medical ICU at the University Hospital Cologne (UHC), Germany, and five medical or surgical ICUs at Columbia University Medical Center (CUMC), New York, United States. A stepwise analytical approach was implemented to systematically evaluate multi-omics integration.

Initially, patients from both institutions were combined into a core dataset (*n* = 88; 64 UHC and 24 CUMC) to assess the baseline impact of incorporating intestinal microbiome and urinary metabolome data on SI prediction. Subsequently, a more detailed investigation was performed on an UHC-only subset comprising 80 patients total (the original 64 core patients plus 16 additional unique UHC patients). From this expanded cohort, 55 patients with complete microbiome and urinary metabolome data were included in integrated predictive modeling. This subset enabled more comprehensive microbiome profiling, including extended characterization of the intestinal microbiome, and respiratory microbiota profiling via bronchoalveolar lavage (BAL) fluid and endotracheal aspirate (ETA) sampling (additional details on dataset characterization are available in Supplementary Figure S1, Additional File 1).

### Patient selection criteria

Eligibility criteria included age ≥ 18 years, ICU admission, and mechanical ventilation initiated within 3 days of enrollment. Exclusion criteria, applied during initial cohort recruitment, included COVID-19 at any point during sampling (laboratory limitations, UHC), prior *Clostridioides difficile* infection within 90 days (CUMC), recent bacterial BSI, or recent ICU admissions (both within 30 days, CUMC).

### Data and specimen collection

Baseline demographic, clinical, and laboratory variables were obtained from medical records at enrollment. For the UHC cohort, biospecimens including BAL fluid, ETA, urine, fecal and deep rectal swabs were collected within 48 h of intubation. Given strong concordance between fecal and rectal swab microbiota profiles, results were pooled accordingly [14]. For the CUMC cohort, rectal swabs and urine were collected within 4 h of ICU admission. In the core dataset, microbiome analysis included assessment of intestinal microbial diversity using the Shannon diversity index [15]. For the subset of UHC patients, analyses were expanded to include respiratory specimens (BAL and ETA) and more detailed microbial profiling beyond standard diversity metrics.

### Outcome definition

SI was defined as new-onset BSI and/or VAP, occurring at least 48 h following enrollment. This threshold is consistent with the Robert Koch Institute (RKI) guideline on nosocomial pneumonia, which defines hospital-acquired pneumonia (including VAP) as pneumonia developing more than 48 h after hospital admission [16]. Diagnostic criteria were further informed by the United States Centers for Disease Control and Prevention (CDC) clinical guidelines for both VAP and BSI cases [17, 18]. All bloodstream cultures were independently reviewed by experienced clinicians to differentiate true bacteremia from contamination; single-positive cultures without supporting clinical evidence were not classified as infections. For each patient, only the first ICU admission was examined, with all SIs captured within 30 days of enrollment.

### Descriptive statistics

Statistical analyses were performed using R (v4.4.1 and v4.2.0; R Foundation, Vienna, Austria) [19]. Continuous variables are presented as medians or means [interquartile range (IQR); full range], and categorical variables as frequencies [%]. Group comparisons used Wilcoxon rank-sum (exact), Fisher’s exact, or Pearson’s chi-squared test, with *p* values < 0.05 considered statistically significant. Multiple testing correction using the Benjamini–Hochberg procedure to control the false discovery rate (FDR) was applied to clinical, microbiome, and metabolite data where appropriate, using a *q* value threshold of 0.05. Random forest imputation addressed selected data missing at random (see Supplementary Table S5 , Additional File 5).

### Microbiome analysis

Microbiome profiling was performed independently at both study sites following established protocols. Methods used for the CUMC cohort have been described previously [13]. For the UHC cohort, details are provided in the Additional File 3; briefly, genomic DNA was extracted using the ZymoBIOMICS™ DNA/RNA Miniprep Kit (Zymo Research; Irvine, CA, USA), and the V3‒V4 regions of the 16 S rRNA gene were sequenced on an Illumina MiSeq platform (Illumina; San Diego, CA, USA). Sequencing data were processed via the DADA2 plugin [20] in QIIME2 [21]. Taxonomic classification was performed via a naïve Bayes classifier (sklearn) [22] trained on the SILVA database release 138 [23]. Microbial dominance was defined as at least 30% relative abundance of a single taxon. Alpha-diversity (α-diversity) was assessed using the Shannon [15] and Simpson’s diversity indices [24], the abundance-based coverage estimator (ACE) [25], the Chao1 estimator [26], species richness (SR), and phylogenetic diversity (PD) [27]. Beta-diversity (β-diversity) was evaluated via UniFrac [28] and Bray‒Curtis dissimilarity [29] metrics. Differential abundance analysis was performed using linear discriminant analysis (LDA) effect size (LEfSe) [30], considering taxa with an LDA score above 2.

### Urine analysis

Urine analysis procedures are detailed in the Additional File 4; briefly, analyses were performed using nuclear magnetic resonance (NMR) spectroscopy and ultra-high performance liquid chromatography-tandem mass spectrometry (UHPLC‒MS/MS). The former was carried out on a Bruker Avance III HD 600 MHz spectrometer, with metabolite identification and quantification performed using the Chenomx NMR Suite 8.3 (Chenomx Inc., Edmonton, Alberta, Canada). Data were processed with TopSpin 4.14 (Bruker) and AMIX 3.9.13 (Bruker). For classification analyses, spectra were divided into 1029 evenly spaced buckets of 0.01 ppm width, followed by creatinine normalization. Spectral overlap in the NMR data has limited interpretability, with some features reflecting multiple compounds. Creatinine and 3-IS were quantified by UHPLC-MS/MS using an ExionLC-30AD HPLC system coupled with a Triple Quad 6500 + mass spectrometer (AB Sciex, Germany, Darmstadt). 3-IS concentration was normalized to creatinine; quantification was based on calibration curves using stable isotope-labeled standards.

### Metabolomic bucket selection

To address dataset heterogeneity, NMR spectral bucket selection was performed using three complementary feature selection approaches: feature importance based on machine learning algorithms (Random Forest, xgbTree, glmnet), selection by filtering based on random forest, and Boruta. The resulting feature set was refined by combining outputs and removing redundancy. Details can be found in the Additional File 4.

### Predictive modeling

To systematically assess the additive value of multi-omics integration, integrative modeling evaluated microbiome and metabolome features through four sequential layers: [1] clinical data alone [2], clinical data combined with microbiome data [3], clinical data combined with selected metabolomic buckets, and [4] all three combined. Candidate predictors included demographic, clinical, comorbidity and severity-of-illness variables (see Additional File 5 for details), along with Shannon diversity, *Enterococcus* dominance (UHC subset only), and selected urinary metabolomic buckets. Covariate selection was performed via stepwise logistic regression in forward, backward, and bidirectional modes, informed by model performance metrics including the Akaike information criterion (AIC) and Nagelkerke’s pseudo-R². The final model refinement was guided by manual review and expert consensus. Collinearity among candidate predictors was systematically assessed via variance inflation factor (VIF) analysis, with predictors exhibiting VIF > 5 excluded to ensure coefficient stability and interpretability.

### Classification models

Machine learning classification models were developed by training (80%) and testing (20%) splits, with recursive feature elimination (RFE) identifying optimal feature subsets, ensuring class balance and avoiding reuse of data between training and testing. Again, four data configurations were tested: [1] clinical data alone [2], clinical data combined with microbiome data [3], clinical data combined with selected metabolomic buckets, and [4] all three combined (candidate predictors detailed in Additional File 5). The models were trained using 5-fold cross-validation repeated five times. Model performance was evaluated via the Matthews correlation coefficient (MCC) [31], and the receiver operating characteristic (ROC) curve with area under the curve (AUC) [32].

### Survival analysis

Survival estimates were calculated using the Kaplan‒Meier method with log-rank testing. Overall survival spans from enrollment to death from any cause, with survivors censored at the time of last contact.

## Results

### Patient characteristics

The core dataset comprised 88 patients, of whom 25 (28%) developed an SI at a median of 11 days following enrollment. The median age was 60 years; 45 patients (52%) were male. The UHC subset comprised 80 patients, with 23 (28%) developing an SI. The median age was 58 years; 46 patients (57%) were male. Demographics were broadly similar across data sets and groups; however, prior antibiotic exposure significantly associated with SI development in both datasets (core dataset: 21 [84%] vs. 26 [41%], *q* = 0.01; UHC subset: 18 [78%] vs. 19 [33%], *q* < 0.01). In the UHC subset, SI patients demonstrated a higher prevalence of hemato-oncological comorbidities (19 [83%] vs. 28 [49%], *q* = 0.045), along with increased use of immunosuppressives (11 [48%] vs. 9 [16%], *q* = 0.02) and chemotherapy (11 [48%] vs. 8 [14%], *q* = 0.01). These patients further exhibited significantly lower platelet counts (75 vs. 180 × 10^9^/L, *q* = 0.046), hemoglobin (8.7 vs. 11.1 g/dL, *q* < 0.01), and hematocrit levels (23% vs. 31%, *q* < 0.01) (see Table 1 and Supplementary Table S1, Additional File 1 for details on the UHC subset).

**Table 1:** Patient characteristics demographic and clinical characteristics of patients included in the core dataset. Comparative summary of the overall dataset and subgroups stratified by secondary infection (SI) status

	Total dataset (*n* = 88) *n* (%), median (IQR)	Patients without SI (*n* = 63) *n* (%), median (IQR)	Patients with SI^a^ (*n* = 25) *n* (%), median (IQR)	*p* value^b^
**Age** (years)	60 (47, 71)	62 (51, 72)	55 (42, 66)	0.56
**Male gender**	45 (52%)	34 (54%)	11 (46%)	1.00
**ICU admission characteristics**				
***Origin***				**0.04**
In-house hospital ward	28 (33%)	14 (23%)	14 (58%)	
Emergency department	39 (46%)	34 (56%)	5 (21%)	
External hospital	18 (21%)	13 (21%)	5 (21%)	
***Admission diagnosis*** ^c^				
Respiratory failure	60 (68%)	43 (68%)	17 (68%)	1.00
Sepsis	22 (25%)	15 (24%)	7 (28%)	1.00
Shock	13 (15%)	11 (17%)	2 (8.0%)	0.91
Neurological condition	13 (15%)	9 (14%)	4 (16%)	1.00
Cardiovascular condition	12 (14%)	10 (16%)	2 (8.0%)	1.00
Other	12 (14%)	9 (14%)	3 (12%)	1.00
**Antibiotic treatment preceding ICU admission** ^d^	47 (53%)	26 (41%)	21 (84%)	**0.01**
**Vasopressor administration at baseline**	80 (92%)	58 (92%)	22 (92%)	1.00
**Administration of anti-infective agents up to day 90**	88 (100%)	63 (100%)	25 (100%)	-
**Clinical and laboratory data at baseline**				
Platelet count [x 10^9^/L]	168 (75, 269)	180 (91, 270)	109 (62, 243)	0.75
Creatinine [mg/dL]	1.2 (0.8, 2.1)	1.1 (0.8, 2.1)	1.3 (0.8, 2.0)	1.00
Bilirubin [mg/dL]	0.6 (0.3, 1.1)	0.6 (0.3, 1.0)	0.9 (0.3, 2.7)	0.56
MAP [mmHg]	73 (63, 80)	70 (61, 80)	75 (65, 80)	1.00
p_a_O_2_ [mmHg]	98 (76, 122)	99 (79, 126)	91 (74, 116)	0.75
F_i_O_2_ [%]	43 (36, 60)	45 (37, 60)	40 (36, 50)	1.00

^a^ UHC: 17 patients; CUMC: 8 patients

^b^ Wilcoxon rank sum test, Pearson’s chi-squared test, Fisher’s exact test; false discovery rate correction for multiple testing

^c^ This variable allowed for multiple answers

^d^ Within six months preceding ICU admission, including treatment at the time of admission, coded as a binary variable (yes/no), encompassing both broad- and narrow-spectrum antibiotics

*IQR* interquartile range, *SI* secondary infection, *ICU* intensive care unit, *MAP* mean arterial pressure, *p*<0.05 significant values are in bold

### Secondary infection characteristics

SIs occurred in 28% (25/88) of patients, 17% (15/88) experienced at least one episode of BSI and 16% (14/88) developed VAP; these categories were not mutually exclusive. The most common BSI pathogens were *Enterococcus* species, partly including vancomycin-resistant strains, and coagulase-negative *Staphylococcus* species. VAP was caused primarily by Gram-negative organisms, including *Klebsiella pneumoniae* (partly multidrug-resistant), *Pseudomonas aeruginosa*, and extended-spectrum beta-lactamase (ESBL)-producing *Escherichia coli* (see Supplementary Table S2, Additional File 2).

### Survival outcomes

In the core dataset, median follow-up extended to 90 days; SI did not significantly impact overall mortality (34% mortality, *p* = 0.79). With more data available, the UHC subset (43% mortality, *p* = 0.09) showed significantly lower ICU and hospital survival among SI patients (*p* = 0.048 and *p* < 0.01, respectively), along with prolonged ICU (33 vs. 11 days) and hospital stays (69 vs. 18 days) (both *p* < 0.01; see Supplementary Figures S7 and S8, Tables S7 and S8, Additional File 6).

### The intestinal and lower respiratory Microbiome

**Fig. 1:**
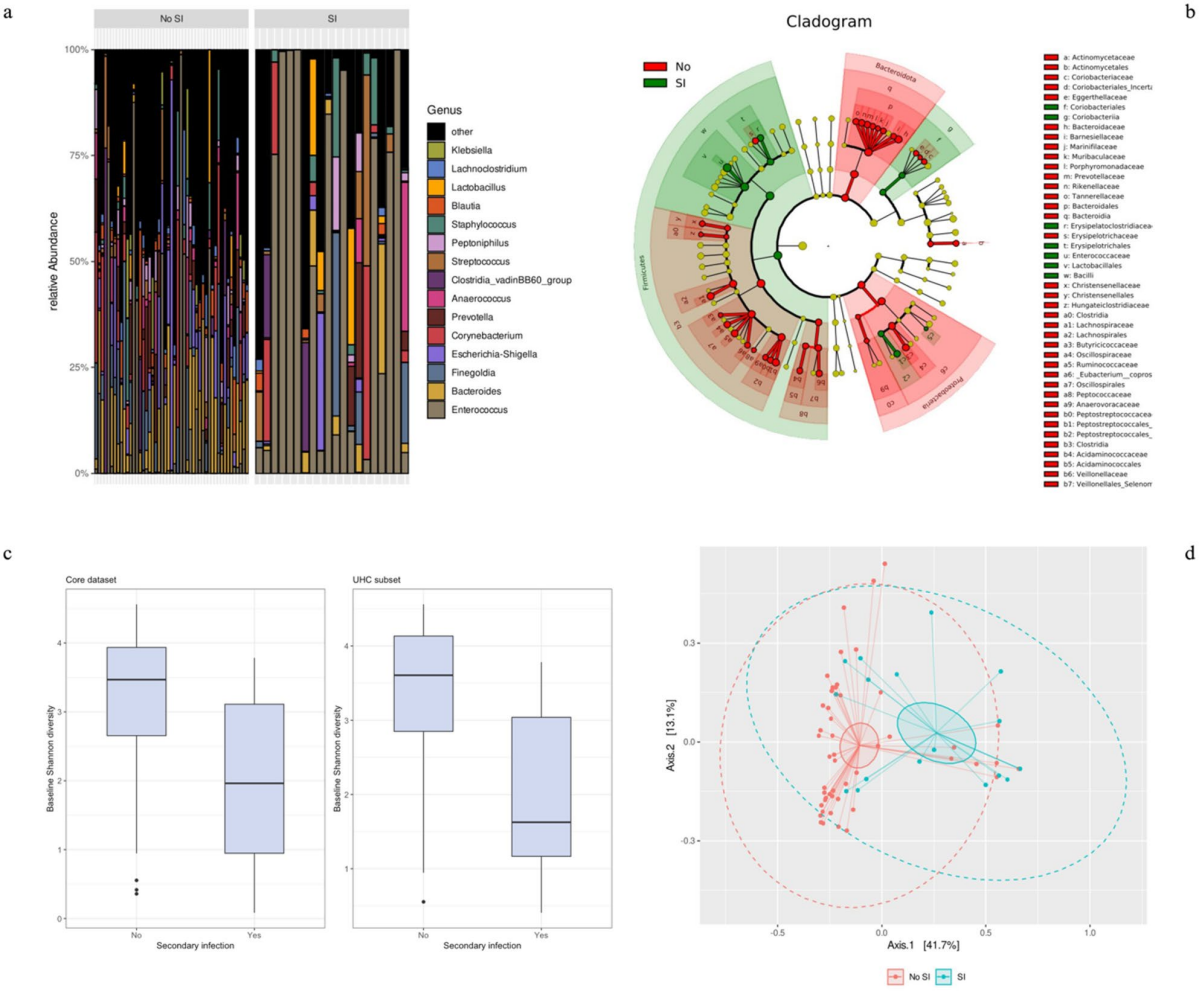
Differences in microbial composition and diversity relative to secondary infection (SI) status (**a**) Genus-level microbial abundance. Bar plots display the relative abundance of the 15 most common bacterial genera identified in patient samples at baseline, comparing individuals without subsequent secondary infection (left panel, No SI) to those with subsequent secondary infection (right panel, SI). Each bar represents one patient, with colors indicating different genera. This visualization provides an overview of the dominant taxa, notably *Enterococcus*, and highlights compositional shifts associated with SI status. Statistical comparisons between groups are presented in Additional File 3, Supplementary Table S3a. (**b**) LEfSe discriminant analysis of microbial taxa. The cladogram summarizes taxa that best distinguish between SI and No SI groups, identified using Linear Discriminant Analysis Effect Size (LEfSe). The circular plot organizes bacteria by phylogenetic relationships, with branch colors marking taxa enriched in either SI (green) or No SI (red) patients. Taxa displayed here contribute most strongly to differences between groups. Corresponding LDA (discriminative effect size) scores are reported in Additional File 3, Supplementary Figure S2b. (**c**) α-diversity comparison. Boxplots show microbial α-diversity (Shannon diversity index) for both the core dataset (left) and the UHC subset (right), stratified by SI status. The Shannon index measures both the richness (number of types) and evenness (distribution among types) of bacteria present within each sample; higher values indicate greater diversity. Statistical results and additional diversity metrics can be found in Additional File 3, Supplementary Table S3c. (**d**) Microbial community structure (β-diversity). Principal Coordinate Analysis (PCoA) plots visualize overall differences in microbial community structure between SI and No SI groups, based on weighted UniFrac distances (which account for both the presence and evolutionary relationships of taxa). Each point represents a patient’s sample; solid ellipses indicate the 95% confidence intervals of the group centroids, while dashed ellipses represent the area expected to contain 95% of the data points for each group, assuming a multivariate normal distribution. The percentage of variation explained by each axis is labeled. Results using alternative distance metrics are presented in Additional File 3, Supplementary Figure S2a

In the core dataset, patients with subsequent SI demonstrated significantly reduced Shannon diversity at enrollment (1.96 vs. 3.47, *p* < 0.01; Fig. 1). In the UHC subset with comprehensive profiling, SI patients exhibited substantially greater baseline *Enterococcus* abundance (46% vs. 11%, *q* < 0.01), with similar patterns at higher taxonomic levels, including the Bacilli class (61% vs. 19%, *q* < 0.01), and Firmicutes phylum domination (70% vs. 22%, *q* < 0.01). In contrast, patients not progressing to SI presented greater relative abundances of anaerobic taxa such as Clostridia (37% vs. 19%, *q* = 0.04), Bacteroidia (20% vs. 7%, *q* < 0.01) and Gammaproteobacteria (12% vs. 4%, *q* = 0.01). LEfSe analysis confirmed the Bacilli class (LDA: 5.286, *p* < 0.01), Lactobacillales order (LDA: 5.258, *p* < 0.01), and Enterococcaceae family (LDA: 5.208, *p* < 0.01) as key discriminatory taxa in SI patients, all within the Firmicutes phylum (see Fig. 1, and Supplementary Table S3 and Figure S2, Additional File 3).

Consistent with the core dataset, microbial diversity was lower in SI cases across all measured α-diversity indices, with significantly reduced Shannon diversity (UHC subset: 1.55 vs. 3.65, *p* < 0.01). β-diversity analyses further delineated compositional segregation between infection and non-infection groups, with weighted UniFrac distance capturing the strongest separation (11.3%, R^2^ = 0.11, pseudo-F = 8.51, *p* < 0.01), indicating that inter-group variance exceeded intra-group variance by over eightfold (see Fig. 1, and Supplementary Table S3 and Figure S2, Additional File 3).

Lower respiratory tract data from the UHC subset showed similar trends toward higher *Enterococcus* species abundance and reduced diversity in SI patients, suggesting parallel microbial disruptions across body sites (see Supplementary Figures S3 and S4, Table S4, Additional File 3).

### Urinary features

**Fig. 2:**
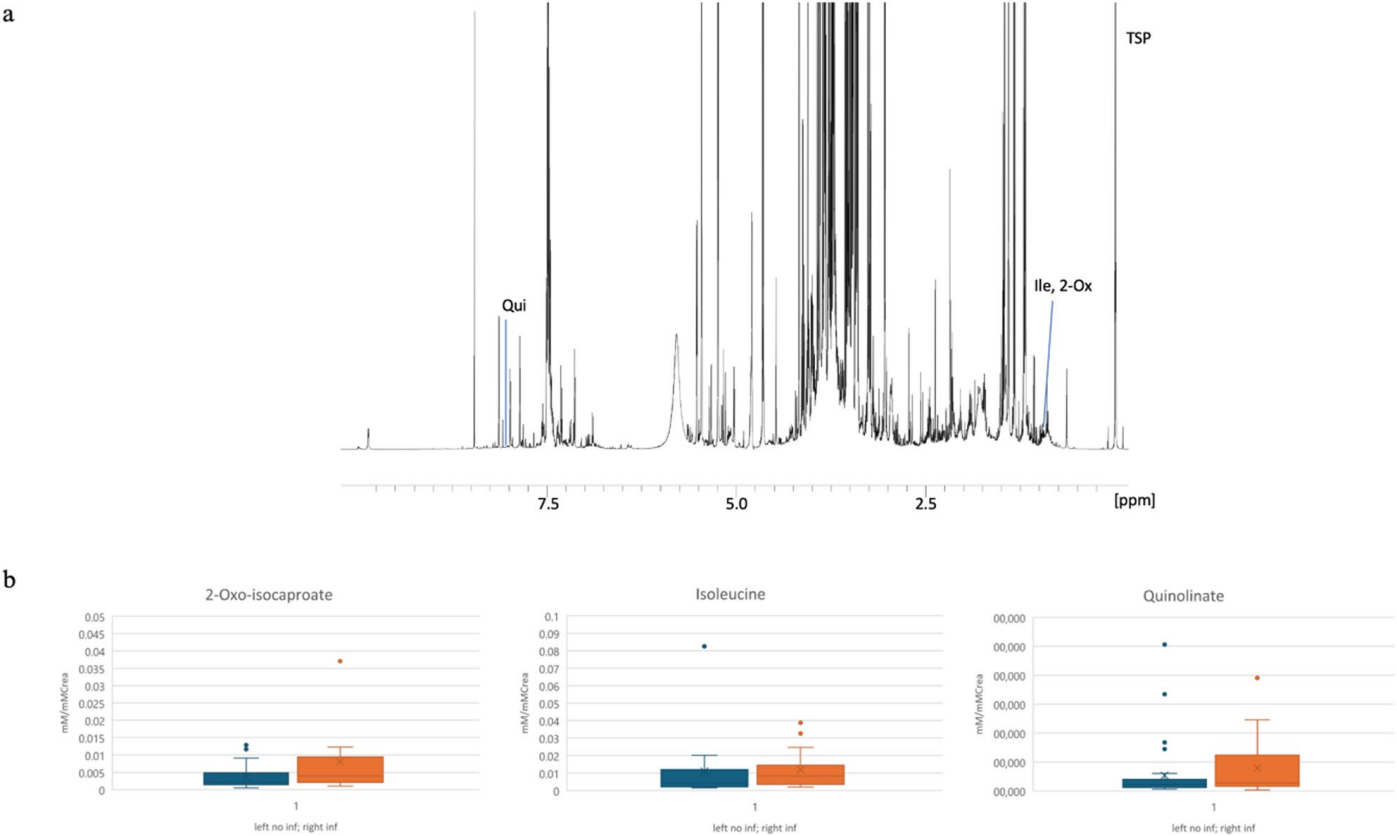
Urinary NMR analysis of metabolites and their creatinine-normalized levels in relation to subsequent secondary infection **(a)** Selected urinary NMR buckets; all buckets have a width of 0.01 ppm; the bucket at 0.935 ppm corresponds to both isoleucine (Ile) and 2-oxoisocaproate (2-Ox), and the bucket at 8.025 ppm corresponds to quinolinate (Qui). **(b)** Creatinine-normalized absolutely quantified levels of 2-oxoisocaproate, isoleucine and quinolinate in patients with and without subsequent secondary infection (inf)

NMR analysis identified several key spectral features associated with SI, as detailed in Additional File 4. Notably, spectral features at 0.925 and 0.935 ppm corresponded primarily to 2-oxoisocaproate and isoleucine, although partial overlap with protein signals was observed. As illustrated in Supplementary Figure S5 of Additional File 4, both 2-oxoisocaproate and, to a lesser degree, isoleucine contribute significantly to the NMR bucket, which comprises signals with chemical shifts ranging from 0.930 to 0.940 ppm. After creatinine normalization, urinary levels of both metabolites were elevated in SI patients relative to non-SI controls, with 2-oxoisocaproate as the predominant component (Fig. 2). Building on previous research [13], 3-IS concentrations and their influence on SI were additionally evaluated but did not differ significantly between groups in the core dataset (see Supplementary Figure S6, Additional File 4).

In the UHC subset, elevated quinolinate (8.025 ppm) associated with SI, whereas 3-IS concentrations were significantly lower in patients with subsequent SI (*p* = 0.01). This latter association, however, did not retain significance in univariate logistic regression (see Fig. 2 and Supplementary Figure S6, Additional File 4).

### Prediction of secondary infection through regression and classification analyses

#### Core dataset analysis

The integrated multi-omics model (clinical data + microbiome data + metabolome data) demonstrated improved performance (AIC = 74.5, R² = 0.53) compared to clinical-only models (AIC = 104.71, R² = 0.10). Key predictors included the urinary NMR feature at 0.935 ppm (odds ratio (OR) = 4.41, 95% confidence interval (CI) 2.18–10.86, *p* < 0.01) and Shannon diversity (OR = 0.35, 95% CI 0.16–0.67, *p* < 0.01). Clinical variables including mean arterial pressure (MAP; OR = 2.12, 95% CI 1.06–4.56, *p* = 0.04) and bilirubin (OR = 1.78, 95% CI 1.05–4.27, *p* = 0.07) further contributed (Table 2).

**Fig. 3:**
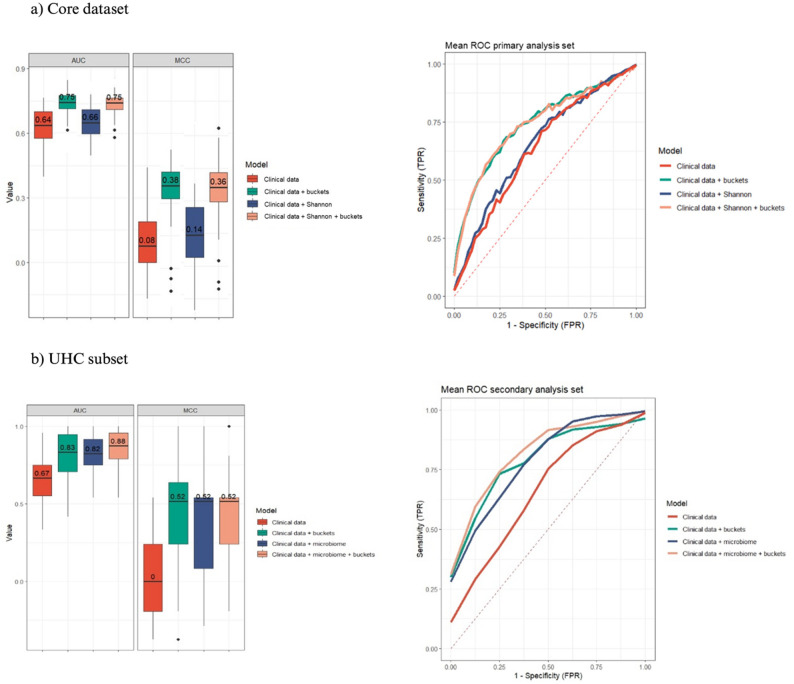
Risk factor analysis for secondary infection. Classification performance results for four data configurations, based on data from the 88 patients in the core dataset (a) and the 55 patients in the UHC subset (b). Left: the box plots illustrate the performance of 50 models across each data configuration on the basis of AUC and MCC scores. Right: the figure shows the mean ROC curves based on the 50 models for each data configuration

Classification analysis showed that microbiome integration (Shannon diversity index) alone yielded marginal improvement (AUC 0.64 to 0.66, ΔAUC = 0.02; MCC 0.08 to 0.14). Metabolomic integration provided substantial gains (AUC 0.64 to 0.75, ΔAUC = 0.11; MCC 0.08 to 0.38). Addition of microbiome data to the metabolomic model conferred no additional benefit (AUC 0.75, MCC 0.36) (Fig. 3).

### UHC subset analysis

Multivariable modeling reinforced findings, with the comprehensive integrated model (AIC = 42.7, R² = 0.61) substantially outperforming clinical-only approaches (AIC = 96.3, R² = 0.07). Key predictors included the Acute Physiology and Chronic Health Evaluation II (APACHE II) score (OR = 3.21, 95% CI 1.26–10.5, *p* = 0.01), Shannon diversity (OR = 0.34, 95% CI 0.11–0.84, *p* = 0.02; Table 2), and *Enterococcus* dominance (OR = 4.96, 95% CI 1.32–19.77, *p* = 0.02 when modeled alone, OR = 2.3, 95% CI 0.4–12.99, *p* = 0.34 when modeled with clinical and metabolome data; Supplementary Table S7, Additional File 5).

Classification analysis demonstrated more substantial microbiome contribution in this dataset: microbiome integration (Shannon diversity index and *Enterococcus* dominance) alone achieved an AUC of 0.82 versus 0.67 for clinical data alone (ΔAUC = 0.15, MCC 0.52). Metabolomic integration similarly yielded an AUC of 0.83 and MCC of 0.52. Concurrent integration of microbiome and metabolome features with clinical variables achieved the highest predictive accuracy (AUC 0.88, MCC 0.52), indicating complementary information across omics layers (Fig. 3).

#### Selected NMR features

The most informative urinary NMR features varied depending on the available clinical and microbiome data, with the 0.935 ppm feature (2-oxoisocaproate and, to a lesser degree, isoleucine) predominating in the core dataset and the 8.025 ppm feature (quinolinate) in the UHC subset (Table 2). However, alternative NMR feature selections yielded comparable predictive performance. Model fit indices were similar across different feature combinations, with AIC values ranging from 74.53 to 88.06 in the core dataset and 41.96 to 50.17 in the UHC subset, indicating that multiple metabolomic signatures could achieve similar discrimination. Detailed feature selection outcomes and model comparisons are presented in Table 2 and Supplementary Table S6 (Additional File 5) (Table 3).

### Antibiotic exposure

When modeled in place of the other clinical parameters from the final models, prior antibiotic exposure emerged as a consistent predictor in the integrated model (core dataset: OR = 2.57, *p* = 0.17; UHC subset: OR = 2.10, *p* = 0.42). VIF analysis confirmed no substantial collinearity with Shannon diversity (VIF = 1.20) and *Enterococcus* dominance (VIF = 1.22), respectively. Nested model comparisons further demonstrate that microbiome features improve predictive performance over models with antibiotic exposure alone (Supplementary Table S6, Additional File 5).

**Table 2:** Multivariable regression analysis for secondary infection, models were constructed using baseline clinical variables alone and in combination with Microbiome and/or urinary metabolome data: core dataset

Characteristic (at baseline)	Clinical data (OR, 95% CI) ^a, b^ AIC = 104.71	Clinical + microbiome data (OR, 95% CI) ^a, c^ AIC = 94.3	Clinical + urinary metabolome data (OR, 95% CI) ^a, d^ AIC = 82.7	Clinical + microbiome + urinary metabolome data (OR, 95% CI) ^a, e^ AIC = 74.5
**Bilirubin**	2.11 (1.13, 5.32; *p* = 0.06)	1.6 (0.96, 3.65; *p* = 0.13)	2.25 (1.2, 5.9; *p* = 0.04)	1.78 (1.05, 4.27; *p* = 0.07)
**MAP**	1.13 (0.68, 1.9; *p* = 0.64)	1.35 (0.21, 0.66; *p* = 0.3)	1.63 (0.89, 3.15; *p* = 0.13)	2.12 (1.06, 4.56; *p* = 0.04)
**Shannon diversity**		0.38 (0.21, 0.66; *p* < 0.01)		0.35 (0.16, 0.67; *p* < 0.01)
**Urinary NMR feature at 0.935 ppm** ^f^			4.33 (2.21, 10.02; *p* < 0.01)	4.41 (2.18, 10.86; *p* < 0.01)

^a^ OR (95% confidence interval, *p* value)

^b^ R^2^ = 0.10

^c^ R^2^ = 0.28

^d^ R^2^ = 0.42

^e^ R^2^ = 0.53

^f^ Key feature identified on the basis of urinary NMR profiles in the core dataset (*n* = 88)

*MAP* mean arterial pressure, *NMR* nuclear magnetic resonance, *ppm* parts per million

**Table 3:** Multivariable regression analysis for secondary infection, models were constructed using baseline clinical variables alone and in combination with Microbiome and/or urinary metabolome data: UHC subset

Characteristic (at baseline)	Clinical data (OR, 95% CI) ^a, b^ AIC = 96.3	Clinical + microbiome data (OR, 95% CI) ^a, c^ AIC = 51.1	Clinical + urinary metabolome data (OR, 95% CI) ^a, d^ AIC = 59.2	Clinical + microbiome + urinary metabolome data (OR, 95% CI) ^a, e^ AIC = 42.7
**APACHE II score**	1.66 (0.99, 2.93; *p* = 0.06)	2.88 (1.33, 7.51; *p* = 0.02)	1.89 (0.96, 3.97; *p* = 0.07)	3.21 (1.26, 10.5; *p* = 0.01)
**Shannon diversity**		0.27 (0.11, 0.58; *p* < 0.01)		0.34 (0.11, 0.84; *p* = 0.02)
**Urinary NMR feature at 8.025 ppm** ^f^			4.57 (2.07, 12.38; *p* < 0.01)	5.11 (1.8, 20.7; *p* < 0.01)

^a^ OR (95% confidence interval (CI), *p* value)

^b^ R^2^ = 0.07

^c^ R^2^ = 0.43

^d^ R^2^ = 0.41

^e^ R^2^ = 0.61

^f^ Key feature identified on the basis of urinary NMR profiles in the subset of UHC patients (*n* = 64)

*APACHE II* Acute Physiology-Age-Chronic Health Evaluation II, *spp.* species, *NMR* nuclear magnetic resonance, *ppm* parts per million

## Discussion

SI remains a significant challenge in the ICU, prolonging critical illness, increasing mortality, and driving up healthcare costs [1, 2]. In this study, SI associated with shorter median survival, reduced overall survival in both ICU and hospital settings, and longer lengths of stay across care environments. The clinical burden of these infections underscores an important gap: despite growing mechanistic insight into host- and pathogen-related determinants of SI, current preventive and diagnostic strategies remain inadequate for early and precise patient risk identification. This work addressed this gap by identifying intestinal microbiome and urinary metabolome features associated with SI development, investigating cross-site microbiota convergence patterns, and determining whether integration of multi-omics data with standard clinical assessment enhances predictive discrimination compared to clinical evaluation alone.

Five key observations emerged from this analysis. First, SI development associated with conventional clinical severity markers, including MAP, bilirubin, and APACHE II scores. Second, intestinal microbiota disruption — characterized by reduced diversity and *Enterococcus* dominance — represented an independent and significant risk factor for SI. Third, antibiotic-induced microbial disruption manifests coordinately across gastrointestinal and respiratory sites. Fourth, distinct urinary metabolomic features mapping to branched-chain amino acids (2-oxoisocaproate, isoleucine) and tryptophan metabolites (quinolinate) emerged as predictors of infection. And fifth, multi-omics integration enhanced predictive accuracy in a context-dependent manner: while metabolomic features consistently improved performance across both datasets, microbiome contributions demonstrated variability dependent upon the specificity of microbial metrics and the dataset employed.

### Clinical risk factors

Established clinical severity indicators retained significant prognostic value for SI prediction in this analysis [33, 34]. MAP and bilirubin, both central components of the Sequential Organ Failure Assessment (SOFA) score [35], as well as the APACHE II score in the UHC subset [36], emerged as consistent predictors. These findings align with the well-established relationship between worsening organ dysfunction and infection susceptibility. In the UHC subset, SI patients presented with a higher prevalence of malignancy, consistent with recognized infection risks in immunocompromised populations [37]. This association likely reflects both the disease burden inherent to underlying malignancy and the established predisposition to poor outcomes characteristic of cancer patients — features often reflected in elevated severity scores [38, 39].

### Microbiome disruption as a key determinant of infection risk

Beyond established clinical factors, microbiome alterations are increasingly recognized as key determinants of adverse outcomes in ICU patients [4]. In our study, baseline *Enterococcus* dominance (≥ 30% relative abundance) significantly associated with infection development, consistent with previous reports linking *Enterococcus* abundance to poor outcomes [6, 7, 40]. This association likely reflects the increased prior antibiotic exposure observed in patients progressing to SI. The ecological mechanisms driving this relationship are well-characterized. Broad-spectrum antibiotics, especially those with anaerobic coverage, disrupt normal microbiome diversity and create ecological niches favorable for *Enterococcus* proliferation, frequently resulting in vancomycin-resistant *Enterococci* (VRE) colonization [40]. Studies have shown that ICU patient microbiota can lose diversity within 72 h of antibiotic exposure, facilitating *Enterococcus* dominance linked to VRE colonization and elevated mortality [6, 7]. Notably, *Enterococcus* dominance at ICU admission has been associated with 19% and 22% increases in mortality and infection risk, respectively, with 77% of such patients exhibiting VRE positivity [7]. In transplant populations, *Enterococcus* dominance increases VRE-derived BSI risk nine-fold [40].

Reduced microbial diversity independently associated with SI development across both study datasets. Higher baseline diversity conferred protective effects, a pattern well-documented in immunocompromised populations [8]. However, traditional diversity metrics may fail to capture critical ecological shifts such as opportunistic pathogen blooms, and their predictive capacity varies with patient population and immune status [6]. In this study, approximately two-thirds of the core dataset originated from a specialized medical ICU focused on hematology-oncology patients, in whom preservation of microbial diversity appeared particularly important. This likely reflects the heightened immune reconstitution needs characteristic of this population; a relationship potentially less pronounced in more heterogeneous ICU settings [7].

Emerging evidence supports the gut‒lung axis as a pathway for microbial translocation and cross-site disruption. In this analysis, respiratory microbiota showed parallel patterns of increased *Enterococcus* abundance and reduced diversity in SI patients, paralleling intestinal patterns. These likely antibiotic-induced ecological shifts contribute to adverse outcomes through loss of colonization resistance across multiple mucosal sites, reinforcing the interdependence of microbial communities in infection susceptibility [41].

### Mechanistic insights into urinary metabolites

Realizing the clinical potential of colonization risk assessment requires reliable, rapidly measurable biomarkers. Urinary metabolites combine the advantages of non-invasive sample collection and compatibility with routine clinical workflows, coupled with its metabolic richness and relative freedom from protein and cellular interference [42–45]. In this study, NMR spectroscopy identified multiple metabolomic features associated with subsequent SI development, with the bucket at 0.935 ppm and 8.025 ppm emerging as the most informative discriminators within final multivariable models. These features were selected in the context of combined clinical and microbiome data, yielding two distinct but biologically plausible signatures across the partially overlapping datasets and underscoring anticipated synergistic interactions among clinical, microbial, and metabolic factors. Importantly, these associations remain correlative rather than causal; shared confounders of critical illness pathophysiology may underlie the observed patterns.

The NMR feature at 0.935 ppm comprises 2‑oxoisocaproate and isoleucine, both closely linked to branched‑chain amino acid (BCAA) metabolism and identified as key discriminators of subsequent SI in the core dataset. Their urinary accumulation likely reflects broader disruptions in host–microbial BCAA metabolism, potentially resulting from depletion of BCAA-consuming taxa, systemic inflammation, or catabolic stress — conditions common in critical illness and frequently accompanied by renal dysfunction. Experimental data suggest that ketoacids such as 2‑oxoisocaproate can modulate macrophage polarization toward pro-tumoral or pro‑inflammatory macrophage states, providing a biologically plausible link between BCAA catabolism and immune function [46]. Elevated isoleucine, in turn, has been associated with chronic metabolic and inflammatory states, including liver disease [47] and type 2 diabetes [48]. It further acts as an immunonutrient that supports immune organ development and function, promotes host‑defense peptide expression, and enhances antimicrobial responses in experimental models [49, 50]. In this context, increased urinary isoleucine in patients who later develop SI may represent a compensatory host response to dysbiosis‑associated or critical‑illness–related immune dysfunction. Although vitamin deficiencies — thiamine [51] and pyridoxine [52] — could theoretically explain elevated levels of 2-oxoisocaproate and isoleucine, routine ICU supplementation and vitamin-enriched nutrition make these etiologies unlikely.

The signal at 8.025 ppm corresponds to quinolinate, a kynurenine pathway intermediate derived from tryptophan degradation, which consistently discriminated SI in the UHC subset. Urinary elevation primarily reflects inflammation-induced activation of host indoleamine 2,3-dioxygenase, the rate-limiting enzyme, driven by proinflammatory cytokines. Reduced microbial tryptophan catabolism by depleted commensals (Clostridia/Bacteroidia), supported by lower 3-IS in SI patients, may secondarily increase substrate availability for this pathway. Functionally, quinolinate serves as a central intermediate in the de novo synthesis of nicotinamide adenine dinucleotide (NAD⁺), essential for cellular energy metabolism, DNA repair, and stress response regulation. During immune activation, increased quinolinate may reflect a metabolic shift toward NAD⁺ production to support host defense, while reduced quinolinate phosphoribosyltransferase activity, impairing conversion to downstream precursors like nicotinic acid mononucleotide, likely promotes accumulation under inflammatory conditions [53–55]. Clinically, elevated quinolinate has been linked to adverse outcomes [56] and neurotoxic, pro-inflammatory effects in other infectious and inflammatory settings, supporting its relevance as a host-response marker [57].

Notably, 2-oxoisocaproate, isoleucine, and quinolinate are largely host-derived metabolites, with microbial communities modulating their levels at varying degrees. This indicates that urinary metabolites capture a host-metabolic dimension of SI risk that is not fully reflected by microbiota composition alone, thereby complementing the information contained in microbiome profiles.

### The complementary predictive value of multi-omics integration

Building on these metabolite insights, integration of multi-omics data into predictive modeling consistently demonstrated improvements over traditional clinical models, though the extent and nature of these gains varied substantially with dataset characteristics and omics modalities employed. In the core dataset, microbiome integration (Shannon diversity index alone) yielded only marginal predictive improvement (ΔAUC = 0.02), whereas the more comprehensively characterized UHC subset demonstrated substantial enhancement (ΔAUC = 0.15) when microbiome data were added (including both the Shannon diversity and *Enterococcus* dominance). This discrepancy likely reflects the differential discriminatory capacity of these microbial metrics: while Shannon diversity quantifies overall community richness and evenness [15], it provides limited independent prognostic information. In contrast, mechanistically informative features such as *Enterococcus* dominance confer substantially greater predictive value. These observations are consistent with systematic reviews of lower gut dysbiosis in critical illness, where reductions in α-diversity do not consistently associate with in-hospital mortality, while specific taxa abundance, including *Enterococcus* species, serve as stronger predictors of clinical outcomes [6]. Recent evidence further underscores the advantage of microbiome data integration: autoencoder-based integration of multi-compartmental microbiome data with clinical variables consistently yields superior prediction accuracy, achieving up to 98% accuracy for mortality in lung microbiome analyses, while microbiome taxa alone perform markedly weaker (53–65%) [58]. Similarly, hierarchical machine learning incorporating time-series microbiome and clinical data for predicting growth faltering in preterm infants has demonstrated marked improvements in predictive precision and cost efficiency compared to clinical data alone [59].

Urinary metabolomic integration, by contrast, consistently improved predictive performance in both datasets. The UHC cohort achieved comparable accuracy from either metabolome or microbiome data alone. Crucially, concurrent integration of both omics layers with clinical variables achieved the highest predictive accuracy (AUC 0.88 in UHC subset), indicating complementary information across distinct biological domains. These findings underscore a fundamental principle: metabolomic signatures appear to capture generalizable immunometabolic states and microbiome-related disruptions relevant to infection susceptibility across heterogeneous clinical contexts. Microbiome features, conversely, exhibit context-dependent predictive value varying substantially based on the specificity and biological informativeness of metrics employed.

## Urinary metabolomics emerging clinical applications in critical illness and infection risk

Accumulating evidence supports urinary metabolomics as a feasible and informative approach for stratifying critically ill populations and predicting adverse outcomes. Urinary metabolic profiles have been linked to cardiovascular risk in type 1 diabetes [60] and progression of chronic kidney disease [61]. Further, urinary metabolites mapping to amino acid biosynthesis and glycolipid metabolism associated with immunoglobulin A nephropathy progression (AUCs exceeding 0.9) [62]. In cirrhotic patients, serum and urinary metabolites involved in S-adenosyl methionine and tryptophan metabolism have shown prognostic value for predicting acute kidney injury and dialysis requirement [63]. Similarly, combat-injured patients exhibited associations between urinary lactate, glycine, and 1-methylnicotinamide levels and mortality or renal replacement therapy need [64], supporting the feasibility of urine-based prognostic models in critically ill populations.

More specifically regarding infection risk and aligned with current findings on quinolinate and 3-IS, recent studies demonstrate urinary metabolomics’ capacity to capture alterations in the tryptophan-kynurenine pathway. In hospitalized COVID-19 patients, elevated urinary kynurenine, 3-hydroxykynurenine, and 3-hydroxyanthranilate correlated with systemic inflammation and disease severity [65]. In surgical ICU patients, plasma quinolinic acid demonstrated very good discrimination between non-septic and pre-septic patients, supporting our findings that tryptophan pathway activation predicts infection risk [66]. Gut-derived uremic toxins such as 3-IS have been proposed as candidate biomarkers of organ dysfunction and may reflect microbiome activity relevant to infection susceptibility [13]. In allogeneic stem cell transplant recipients, low urinary 3-IS levels associated with significantly increased transplant-related mortality, reinforcing the protective role of this microbiome-derived metabolite [12]. Emerging work further demonstrates urinary metabolomics’ ability to discriminate pathogen-specific infection phenotypes — unique urinary metabolite patterns distinguish *Pseudomonas aeruginosa* VAP from both non-*Pseudomonas* VAP and uninfected controls [67]. Lastly, urine profiling has identified agmatine and N6-methyladenine as markers accurately predicting urinary tract infection pathogens across large cohorts [68, 69].

### Methodological considerations and limitations

Several constraints warrant explicit acknowledgment as they impact appropriate interpretation and generalizability of our findings. The modest patient numbers (*n* = 88 core dataset, *n* = 55 UHC subset) combined with clinical heterogeneity result in moderate statistical power and an increased risk of overfitting, particularly given the high dimensionality of the NMR metabolomic dataset (1,029 spectral buckets). The results therefore reflect within-cohort performance without formal external validation, limiting confidence in model transportability to other ICU settings.

Post hoc integration of two geographically and clinically distinct cohorts was undertaken to enhance statistical power and generalizability, yet inherently introduces heterogeneity through differences in patient demographics, healthcare systems, clinical practices, infection-control protocols, and specimen collection procedures. These differences may confound biological signals and bias feature selection toward cohort-specific associations, thereby limiting generalizability to other ICU contexts.

From a microbiological perspective, 16 S rRNA gene sequencing provides only genus- to species-level taxonomic resolution, precluding strain-level discrimination. Cross-cohort variations in sequencing workflows introduce batch effects potentially confounding biological interpretation.

Spectral overlap in untargeted NMR metabolomics limits definitive metabolite annotation, such that bucket integrals function as proxies for underlying metabolic pathways. Consequently, the reported associations involving 2-oxoisocaproate, isoleucine, and quinolinate should be interpreted cautiously and independently validated through orthogonal quantitative assays to confirm metabolite identities and enable mechanistic interpretation.

Characterization of antibiotic exposure was limited to clinical documentation without stratification by drug class, dosing, or pharmacokinetics, precluding quantitative assessment of selective pressure and class-specific effects on microbiota composition.

Finally, single baseline sampling provides only a metabolic and microbial snapshot; dynamic alterations preceding sampling or emerging prior to infection onset remain uncharacterized. This design obscures temporal relationships between predictive marker detection and infection development, precluding determination of whether identified signatures represent primary drivers of infection susceptibility or downstream consequences of early dysbiosis. The dynamic trajectory of microbiota-metabolome alterations during the at-risk period remains unexplored.

### Clinical translation requirements

Given these constraints, modeling results should be interpreted as proof-of-concept rather than a ready-for-deployment clinical prediction tool. External validation and further mechanistic investigation are essential prerequisites before clinical translation. Prospective studies must validate findings across larger, more homogeneous patient populations while examining interactions between underlying pathophysiology (e.g., sepsis, acute respiratory distress syndrome, post-surgical critical illness) and dysbiotic trajectories. Ideally, true external validation through training on one cohort and prospective testing on an independent population would rigorously establish model transportability.

Longitudinal profiling at frequent intervals during early ICU admission will enable trajectory analysis determining whether progressive microbiome disruption predicts infection development with greater sensitivity than baseline assessment. Emerging evidence suggests that biomarker kinetics over time confer superior prognostic value compared to single-timepoint measurements [13]; our preliminary data reveal microbiome convergence patterns as early as enrollment, emphasizing the critical importance of temporal resolution in capturing dysbiotic trajectories. Extending this longitudinal approach to evaluate the influence of antibiotic exposure on microbiome evolution will be essential to differentiate treatment effects from intrinsic disease dynamics. Furthermore, comparing recovery trajectories in successfully treated versus secondarily infected patients may help identify dynamic microbiome and metabolome markers with higher predictive potential than static measurements.

Mechanistic validation remains essential for translational impact. Targeted quantitative assays should confirm associations with 2-oxoisocaproate, isoleucine, and quinolinate, establishing absolute concentrations and validating amino acid metabolism dysregulation as a microbiome-derived mechanism of infection susceptibility. The differential selection of NMR features between datasets further suggests context-dependence of metabolomic signatures, warranting systematic exploration of geographic and clinical variations in biomarker selection across cohorts to clarify whether universal signatures exist or whether context-specific markers are required for optimal performance.

A critical recognition must frame all future work: both 16 S rRNA sequencing and NMR metabolomics remain research tools unsuitable for real-time clinical decision-making. Sequencing requires a few hours up to days for sample processing and analysis; NMR similarly demands substantial analytical infrastructure and technical expertise. Bridging this technology gap demands development of rapid, point-of-care assays that preserve the mechanistic insights identified herein while meeting practical ICU requirements: minimal specimen handling, turnaround time of hours rather than days, cost-effectiveness permitting routine deployment, and interpretability for clinical personnel without specialist training.

## Conclusion

This systematic evaluation of multi-omics integration provides modest but consistent evidence for the added value of incorporating intestinal microbiome and urinary metabolome data with standard clinical assessment for SI prediction in critically ill patients. SI susceptibility associates with clinical severity markers, microbiota disruption (particularly diminished microbial diversity and *Enterococcus* dominance), and dysregulated amino acid and tryptophan metabolism reflected in urinary metabolite alterations. Multi-omics integration enhanced predictive discrimination dependent upon dataset and metric specificity.

These findings establish a foundation for prospective validation studies and mechanistic investigation, bringing closer a future precision medicine approach to infection prevention in the ICU. However, substantial research and technical development remain necessary before these biomarkers and analytical approaches transition from research tools to clinically deployable interventions.

## Supplementary Information

Below is the link to the electronic supplementary material.

Supplementary Material 1: Patient enrollment, study design, and clinical characteristics of the UHC subset.

Supplementary Material 2: Secondary infection characteristics.

Supplementary Material 3: Microbiome analyses and corresponding extended findings.

Supplementary Material 4: Urine analyses and corresponding extended findings.

Supplementary Material 5: Classification analysis, missing data, and extended findings of the multivariable regression analysis.

Supplementary Material 6: Survival analysis.

## Data Availability

The datasets used and/or analyzed during the current study are available from the corresponding author upon reasonable request.

## Abbreviations

3-IS: 3-indoxyl sulfate
ACE: Abundance-based coverage estimator
AIC: Akaike information criterion
APACHE II: Acute Physiology-Age-Chronic Health Evaluation II
AUC: Area under the curve
BAL: Bronchoalveolar lavage
BCAA: Branched-chain amino acid
BSI: bloodstream infection
CDC: Centers for Disease Control and Prevention
CUMC: Columbia University Medical Center
ESBL: Extended-spectrum beta-lactamase
ETA: Endotracheal aspirates
FDR: False discovery rate
ICU: Intensive care unit
IQR: Interquartile range
LDA: Linear discriminant analysis
LEfSe: Linear discriminant analysis effect size
MAP: Mean arterial pressure
MCC: Matthews correlation coefficient
NAD^+^: Nicotinamide adenine dinucleotide
NMR: Nuclear magnetic resonance
OR: Odds ratio
PCoA: Principal coordinate analysis
PD: Phylogenetic diversity
RFE: Recursive feature elimination
RKI: Robert Koch Institute
ROC: Receiver operating characteristic
SI: Secondary infection
SOFA: Sequential Organ Failure Assessment
SR: Species richness
UHC: University Hospital Cologne
UHPLC‒MS/MS: Ultra-High Performance Liquid Chromatography‒Tandem Mass Spectrometry
VAP: Ventilator-associated pneumonia
VIF: Variance inflation factor
VRE: Vancomycin-resistant *Enterococci*
