# Supplementary material for: Integrating intestinal microbiome and urinary metabolome data to predict secondary infection in critically ill patients
Source: Crit Care. 2026 Mar 13;30:161. doi: 10.1186/s13054-025-05818-5 (PMC13064364; doi:10.1186/s13054-025-05818-5)
Supplement: Supplementary file 1 — Supplementary Material 1: Patient enrollment, study design, and clinical characteristics of the UHC subset. [file 13054_2025_5818_MOESM1_ESM.docx]

**Integrating intestinal microbiome and urinary metabolome data**

**to predict secondary infection in critically ill patients**

**Critical Care**

Charlotte Linz^1^, Kristiyana Tsenova^2^, Katja Dettmer^3^, Lisa Ellmann^3^, Peter J. Oefner^3^

Wolfram Gronwald^3^, Fedja Farowski^1,2^, Alina M. Rüb^1,2^, Daniel E. Freedberg^4^, Philipp Koehler^1,5,6^

Jorge Garcia Borrega^1^, Jan-Hendrik Naendrup^1^, Maria J.G.T. Vehreschild^1,2^ * and Boris Böll^1+^ *

* Contributed equally

^1^ University of Cologne, Cologne, Germany, Faculty of Medicine and University Hospital Cologne, Department I of Internal Medicine, Division of Hematology-Oncology/Critical Care Medicine/Infectious Diseases, Center for Integrated Oncology Aachen Bonn Cologne Düsseldorf (CIO ABCD)

^2^ Goethe University Frankfurt, Frankfurt am Main, Germany, University Hospital Frankfurt, Department II of Internal Medicine, Infectious Diseases

^3^ University of Regensburg, Regensburg, Germany, Institute of Functional Genomics

^4^ Columbia University, New York, United States, Division of Digestive and Liver Diseases, Mailman School of Public Health, Department of Epidemiology

^5^ University of Cologne, Cologne, Germany, Faculty of Medicine and University Hospital Cologne, Department I of Internal Medicine, Division of Clinical Immunology

^6^ University of Cologne, Cologne, Ger­many, Faculty of Medicine and University Hospital Cologne, Institute of Translational Research, Cologne Excellence Cluster on Cellular Stress Responses in Aging-Associated Diseases (CECAD)

**+** Correspondence: Boris Böll, University Hospital Cologne, Kerpener Strasse 62, Cologne, Germany, email: boris.boell@uk‑koeln.de

Additional File 1: patient enrollment, study design, and clinical characteristics of the UHC subset

Additional File 2: secondary infection characteristics

Additional File 3: microbiome analyses and corresponding extended findings

Additional File 4: urine analyses and corresponding extended findings

**Additional File 5: classification analysis, missing data, and extended findings of the multivariable regression analysis**

Additional File 6: survival analysis

**Classification models**

Machine learning and classification models were developed using training (80%) and testing (20%) splits, with multiple random seeds to ensure robust evaluation. RFE with 50 iterations, implemented via caret::rfeControl and a Random Forest model, identified optimal feature subsets (5–20 features).

Four data configurations were tested: (1) clinical data alone, (2) clinical data with Shannon diversity of microbiome composition, (3) clinical data with selected buckets from the NMR-based urinary metabolome, and (4) all three combined.

The Matthews Correlation Coefficient (MCC) was used to evaluate the performance of binary classification mod­els. It is defined as:

$$MCC=\frac{(TP \cdot TN)-(FP \cdot FN)}{\sqrt{(TP + FP)(TP + FN)(TN + FP)(TN + FN)}}$$

where TP, TN, FP, and FN are the true positive, true negative, false positive, and false negative counts, respec­tively. The Receiver Operating Characteristic (ROC) curve is a graphical representation of the diagnostic ability of a binary classifier, plotting the true positive rate (sensitivity) against the false positive rate (1-specificity) at various threshold levels. The Area Under the Curve (AUC) quantifies the overall model performance.

**Missing data**

**Table S5a: Missing data from the core dataset**

All listed clinical and microbiome variables were imputed for the multivariable regression analysis. Metabolite data were complete due to cohort inclusion criteria.

| **Variable** | **Missing (n/N)** | **Imputation for regression analysis** |
| --- | --- | --- |
|  |  |  |
| Bilirubin at baseline | 13/88 | Yes |
| Shannon diversity at baseline | 9/88 | Yes |
| Mean arterial pressure at baseline | 6/88 | Yes |
| Sex | 1/88 | Yes |
| Age at baseline | 1/88 | Yes |
| Use of vasopressors at baseline | 1/88 | Yes |
|  |  |  |

**Table S5b: Missing data from the UHC subset**

Clinical variables were imputed as indicated; microbiome and me­tabolite data were not imputed.

| **Variable** | **Missing (n/N)** | **Imputation for regression analysis** |
| --- | --- | --- |
|  |  |  |
| Procalcitonin at baseline | 25/80 | No |
| Bilirubin at baseline | 14/80 | No |
| BMI at baseline | 9/80 | Yes |
| SIRS at baseline | 6/80 | Yes |
| PEEP at baseline | 6/80 | No |
| Horovitz index at baseline | 6/80 | No |
| APACHE II at baseline | 6/80 | Yes |
| Karnofsky Performance Scale Index at baseline | 5/80 | Yes |
| ECOG Performance Status Scale at baseline | 5/80 | Yes |
| Tidal volume at baseline | 5/80 | No |
| Max. respiratory rate at baseline | 5/80 | No |
| Core-10-TISS at baseline | 4/80 | Yes |
| SAPS II at baseline | 4/80 | Yes |
| qSOFA at baseline | 4/80 | Yes |
| Max. temperature at baseline | 4/80 | No |
| Max heart rate at baseline | 4/80 | No |
| Mean arterial pressure at baseline | 4/80 | No |
|  |  |  |

**Candidate predictor variables for multivariable modeling**

***Core dataset***

- **Demographics**
  - Age [years]
  - Sex (male/female)
- **ICU admission route** [in-house hospital ward, emergency department, external hospital]
- **ICU admission diagnoses** *(all coded as binary variables)*
  - Respiratory failure [yes/no]
  - Sepsis [yes/no]
  - Shock [yes/no]
  - Cardiovascular condition [yes/no]
  - Neurological condition [yes/no]
- **Initial treatment at baseline**
  - Vasopressor use [yes/no]
- **Clinical and laboratory data at baseline**
  - Mean arterial pressure (MAP) [mmHg]
  - Arterial partial pressure of oxygen (PaO₂) [mmHg]
  - Fraction of inspired oxygen (FiO₂) [%]
  - Serum creatinine [mg/dL]
  - Platelet count [x 10⁹/L]
  - Bilirubin [mg/dL]
- **Pre-hospital exposure**
  - Prior antibiotic treatment [yes/no]
    *(defined as antibiotic use within 6 months prior to ICU admission, including ongoing treat­ment at admission; includes both broad- and narrow-spectrum antibiotics)*
- **Microbiome feature at baseline**
  - Intestinal Shannon diversity index [unitless]
- **Metabolome features (urinary NMR spectral buckets)**
  - NMR feature at 0.925 ppm [relative intensity]
  - NMR feature at 0.935 ppm [relative intensity]
  - NMR feature at 0.945 ppm [relative intensity]
  - NMR feature at 2.015 ppm [relative intensity]
  - NMR feature at 2.025 ppm [relative intensity]
  - NMR feature at 2.765 ppm [relative intensity]
  - NMR feature at 2.775 ppm [relative intensity]
  - NMR feature at 3.145 ppm [relative intensity]

***UHC subset***

- **Demographics**
  - Age [years]
  - Sex (male/female)
  - BMI [kg/m²]
- **Performance status at baseline**
  - Eastern Cooperative Oncology Group (ECOG) score [points]
- **ICU admission diagnoses** (all coded as binary variables)
  - Respiratory failure [yes/no]
  - Cardiovascular condition [yes/no]
  - Neurological condition [yes/no]
  - Shock [yes/no]
- **Comorbidities** (all coded as binary variables)
  - Cardiovascular [yes/no]
  - Hemato-oncological [yes/no]
  - Gastrointestinal [yes/no]
  - Endocrine [yes/no]
  - Pulmonary [yes/no]
  - Neurological [yes/no]
  - Renal [yes/no]
  - None [yes/no]
- **Underlying conditions** (binary)
  - Prior to baseline
    - Allogeneic stem cell trans­plant [yes/no]
    - Organ transplant [yes/no]
    - Hereditary immunodeficiency [yes/no]
  - At baseline
    - Systemic inflammatory response syndrome (SIRS) [yes/no]
    - Sepsis [yes/no]
- **Pre-hospital exposure**
  - Prior antibiotic treatment [yes/no]

*(defined as antibiotic use within 6 months prior to ICU admission, including ongoing treat­ment at admission; both broad- and narrow-spectrum antibiotics included)*

- **Illness Severity scores and comorbidity indices at baseline**
  - Charlson Comorbidity Index (CCI) [points]
  - qSOFA score [points]
  - APACHE II score [points]
  - Core-10-TISS score [points]
  - SAPS II score [points]
- **Microbiome features at baseline**
  - Intestinal Shannon diversity index [unitless]
  - *Enterococcus* spp. dominance [yes/no]
- **Metabolome features (urinary NMR spectral buckets)**
  - NMR feature at 1.029 ppm [relative intensity]
  - NMR feature at 2.015 ppm [relative intensity]
  - NMR feature at 4.555 ppm [relative intensity]
  - NMR feature at 8.025 ppm [relative intensity]

**Candidate predictor variables for classification models**

***Core dataset***

- **Demographics**
  - Age [years]
  - Sex (male/female)
- **ICU admission route** [in-house hospital ward, emergency department, external hospital]
- **ICU admission diagnoses** *(all coded as binary variables)*
  - Respiratory failure [yes/no]
  - Sepsis [yes/no]
  - Shock [yes/no]
  - Cardiovascular condition [yes/no]
  - Neurological condition [yes/no]
  - Other [yes/no]
- **Initial treatment at baseline**
  - Vasopressor use [yes/no]
- **Clinical and laboratory data at baseline**
  - Mean arterial pressure (MAP) [mmHg]
  - Arterial partial pressure of oxygen (PaO₂) [mmHg]
  - Fraction of inspired oxygen (FiO₂) [%]
  - Serum creatinine [mg/dL]
  - Platelet count [10⁹/L]
  - Bilirubin [mg/dL]
- **Pre-hospital exposure**
  - Prior antibiotic treatment [yes/no]
    *(defined as antibiotic use within 6 months prior to ICU admission, including ongoing treat­ment at admission; includes both broad- and narrow-spectrum antibiotics)*
- **Microbiome feature at baseline**
  - Intestinal Shannon diversity index [unitless]
- **Metabolome features (urinary NMR spectral buckets)**
  - NMR feature at 0.925 ppm [relative intensity]
  - NMR feature at 0.935 ppm [relative intensity]
  - NMR feature at 0.945 ppm [relative intensity]
  - NMR feature at 2.015 ppm [relative intensity]
  - NMR feature at 2.025 ppm [relative intensity]
  - NMR feature at 2.765 ppm [relative intensity]
  - NMR feature at 2.775 ppm [relative intensity]
  - NMR feature at 3.145 ppm [relative intensity]

***UHC subset***

- **Demographics**
  - Sex (male/female)
  - BMI [kg/m²]
- **Performance status at baseline**
  - Eastern Cooperative Oncology Group (ECOG) score [points]
- **ICU admission diagnoses** (all coded as binary variables)
  - Respiratory failure [yes/no]
  - Sepsis [yes/no]
  - Cardiovascular condition [yes/no]
  - Neurological condition [yes/no]
  - Shock [yes/no]
- **Comorbidities** (all coded as binary variables)
  - Cardiovascular [yes/no]
  - Hemato-oncological [yes/no]
  - Gastrointestinal [yes/no]
  - Endocrine [yes/no]
  - Pulmonary [yes/no]
  - Neurological [yes/no]
  - Renal [yes/no]
  - None [yes/no]
- **Underlying conditions** (binary)
  - Prior to baseline
    - Allogeneic stem cell trans­plant [yes/no]
    - Organ transplant [yes/no]
    - Hereditary immunodeficiency [yes/no]
  - At baseline
    - Systemic inflammatory response syndrome (SIRS) [yes/no]
    - Sepsis [yes/no]
- **Pre-hospital exposure**
  - Prior antibiotic treatment [yes/no]

*(defined as antibiotic use within 6 months prior to ICU admission, including ongoing treat­ment at admission; both broad- and narrow-spectrum antibiotics included)*

- **Illness Severity scores and comorbidity indices at baseline**
  - Charlson Comorbidity Index (CCI) [points]
  - qSOFA score [points]
  - APACHE II score [points]
  - Core-10-TISS score [points]
  - SAPS II score [points]
- **Clinical and laboratory data at baseline**
  - Mean arterial pressure (MAP) [mmHg]
  - White blood cell count (WBC) [x 10^9^/L]
  - Platelet count [x 10⁹/L]
  - Hematocrit [%]
  - Hemoglobin [g/dL]
  - Serum creatinine [mg/dL]
  - Bilirubin [mg/dL]
  - Glutamic-oxaloacetic transaminase (GOT) [U/L]
  - Glutamic-pyruvic transaminase (GPT) [U/L]
  - C-reactive protein (CRP) [mg/L]
  - Procalcitonin (PCT) [µg/L]
- **Microbiome features at baseline**
  - Intestinal Shannon diversity index [unitless]
  - *Enterococcus* spp. dominance [yes/no]
- **Metabolome features (urinary NMR spectral buckets)**
  - NMR feature at 1.029 ppm [relative intensity]
  - NMR feature at 2.015 ppm [relative intensity]
  - NMR feature at 4.555 ppm [relative intensity]
  - NMR feature at 8.025 ppm [relative intensity]

**Multivariable regression analyses**

**Table S6:** **Regression analysis models using identified urinary metabolomic features and alternative clinical and mi­crobiome features**

**Table S6a: Predictive models for the core dataset including alternative urinary NMR features**

|  | **Urinary NMR feature at 0.925 ppm**^a^ | **Urinary NMR feature at 0.935 ppm**^a^ | **Urinary NMR feature at 0.945 ppm**^a^ | **Urinary NMR feature at 2.015 ppm**^a^ | **Urinary NMR feature at 2.025 ppm**^a^ | **Urinary NMR feature at 2.765 ppm**^a^ | **Urinary NMR feature at 2.775 ppm**^a^ | **Urinary NMR feature at 3.145 ppm**^a^ |
| --- | --- | --- | --- | --- | --- | --- | --- | --- |
|  |  |  |  |  |  |  |  |  |
| **Bilirubin**^b^ | 1.77 (1.04, 4.32; *p*= 0.08) | 1.78 (1.05, 4.27; *p*= 0.07) | 1.75 (1.04, 4.12; *p*= 0.07) | 1.38 (0.79, 3.49; *p*= 0.35) | 1.46 (0.86, 3.51; *p*= 0.24) | 1.45 (0.87 3.21; *p*= 0.2 | 1.48 (0.89 3.25; *p*= 0.18) | 1.47 (0.84, 3.6; *p*= 0.26) |
|  |  |  |  |  |  |  |  |  |
| **MAP**^b^ | 1.93 (0.97, 4.1; *p* = 0.07) | 2.12 (1.06, 4.56; *p* = 0.04) | 2.05 (1.04, 4.33; *p* = 0.046) | 1.66 (0.9, 3.2; *p* = 0.11) | 1.74 (0.94, 3.42; *p* = 0.09) | 1.69 (0.9, 3.26; *p* = 0.1) | 1.63 (0.89, 3.09; *p* = 0.12) | 1.36 (0.75, 2.44; *p* = 0.29) |
|  |  |  |  |  |  |  |  |  |
| **Shannon diversity**^b^ | 0.34 (0.16, 0.65; *p* = 0.002) | 0.35 (0.16, 0.67; *p* = 0.003) | 0.36 (0.17, 0.69; *p* = 0.004) | 0.41 (0.21, 0.74; *p* = 0.005) | 0.39 (0.2, 0.73; *p* = 0.004) | 0.45 (0.23, 0.82; *p* = 0.01) | 0.45 (0.23, 0.81; *p* = 0.01) | 0.44 (0.23, 0.79; *p* = 0.008) |
|  |  |  |  |  |  |  |  |  |
| **0.925 ppm** | 4.2 (2.1, 10.37; *p* = < 0.001) |  |  |  |  |  |  |  |
| **0.935 ppm** |  | 4.41 (2.18, 10.86; *p* = < 0.001) |  |  |  |  |  |  |
| **0.945 ppm** |  |  | 3.71 (1.97, 8.18; *p* = < 0.001) |  |  |  |  |  |
| **2.015 ppm** |  |  |  | 2.53 (1.43, 5.11; *p* = 0.004) |  |  |  |  |
| **2.025 ppm** |  |  |  |  | 2.77 (1.53, 6.11; *p* = 0.003) |  |  |  |
| **2.765 ppm** |  |  |  |  |  | 3.22 (1.64, 7.06; *p* = 0.002) |  |  |
| **2.775 ppm** |  |  |  |  |  |  | 3.56 (1.7, 8.54; *p* = 0.002) |  |
| **3.145 ppm** |  |  |  |  |  |  |  | 2.24 (1.26, 4.95; *p* = 0.02) |
|  |  |  |  |  |  |  |  |  |
| **AIC** | 75.47 | 74.53 | 76.47 | 83.93 | 85.55 | 83.69 | 83.47 | 88.06 |
| **Pseudo-R^2^** | 0.52 | 0.53 | 0.51 | 0.43 | 0.41 | 0.43 | 0.43 | 0.38 |
|  |  |  |  |  |  |  |  |  |

^a^ OR (95% confidence interval, *p* value)

^b^ At baseline

*MAP* mean arterial pressure, *NMR* nuclear magnetic resonance, *ppm* parts per million, *AIC* Akaike information criterion

**Table S6b: Predictive models for the UHC subset including alternative urinary NMR features**

|  | **Urinary NMR feature at 1.029 ppm**^a^ | **Urinary NMR feature at 2.015** **ppm**^a^ | **Urinary NMR feature at 4.555 ppm**^a^ | **Urinary NMR feature at 8.025 ppm**^a^ |
| --- | --- | --- | --- | --- |
|  |  |  |  |  |
| **APACHE II score**^b^ | 2.48 (1.08, 6.8; *p* = 0.05) | 0.61 (0.9, 9.88; *p* = 0.09) | 2.99 (1.34, 8.07; *p* = 0.014) | 3.21 (1.26, 10.5; *p* = 0.013) |
|  |  |  |  |  |
| **Shannon diversity**^b^ | 0.24 (0.07, 0.57; *p* = 0.004) | 0.25 (0.08, 0.61; *p* = 0.006) | 0.322 (0.12, 0.72; *p* = 0.01) | 0.34 (0.11, 0.84; *p* = 0.018) |
|  |  |  |  |  |
| **2.015** **ppm** |  | 4.22 (1.67, 15.98; *p* = 0.012) |  |  |
| **8.025 ppm** |  |  |  | 5.11 (1.8, 20.7; *p* = 0.001) |
| **1.029 ppm** | 3.99 (1.48, 16.86; *p* = 0.03) |  |  |  |
| **4.555 ppm** |  |  | 1.84 (0.92, 4.31; *p* = 0.1) |  |
|  |  |  |  |  |
| **AIC** | 43.869 | 41.962 | 50.165 | 42.657 |
| **Pseudo-R^2^** | 0.59 | 0.62 | 0.48 | 0.61 |
|  |  |  |  |  |

^a^ OR (95% confidence interval, *p* value)

^b^ At baseline

*NMR* nuclear magnetic resonance, *ppm* parts per million, *AIC* Akaike information criterion

**Table S6c:** **Predictive models for the UHC subset including alternative microbiome features**

| **Characteristic**  (at baseline) | **Shannon diversity alone**  (OR, 95% CI) ^a,b^  AIC = 56.7 | ***Enterococcus* spp. dominance alone**  (OR, 95% CI) ^a,c^  AIC = 62.9 | ***Enterococcus* spp. + APACHE II**  (OR, 95% CI) ^a,d^  AIC = 75.4 | **Final model with *Enterococcus* spp. dominance**  (OR, 95% CI) ^a,e^  AIC = 47.4 |
| --- | --- | --- | --- | --- |
|  |  |  |  |  |
| **APACHE II score** |  |  | 1.09 (1.01, 1.19; *p*= 0.03) | 2.64 (1.14, 7.33; *p*= 0.04) |
|  |  |  |  |  |
| **Shannon diversity** | 0.33 (0.16, 0.64; *p*< 0.01) |  |  |  |
|  |  |  |  |  |
| ***Enterococcus* spp. dominance** |  | 4.96 (1.32, 19.77; *p*= 0.02) | 6.16 (1.83, 22.7; *p*< 0.01) | 2.3 (0.4, 12.99; *p*= 0.34) |
|  |  |  |  |  |
| **Urinary NMR feature at 8.025 ppm**^f^ |  |  |  | 5.77 (2.07, 22.87; *p*< 0.01) |
|  |  |  |  |  |

^a^ OR (95% confidence interval (CI), *p* value)

^b^ R^2^ = 0.28

^c^ R^2^ = 0.14

^d^ R^2^ = 0.26

^e^ R^2^ = 0.53

^f^ Key feature identified on the basis of urinary NMR profiles in the subset of UHC patients (n = 64)

*APACHE II* Acute Physiology-Age-Chronic Health Evaluation II, *spp.* species, *NMR* nuclear magnetic reso­nance, *ppm* parts per million, *AIC* Akaike information criterion

**Table S6d: Predictive models including prior antibiotic exposure and Shannon diversity**

| **Characteristic**  (at baseline) | **Prior antibiotic exposure**  (OR 95% CI) ^a^ | **AIC**  **R^2^** | **Prior antibiotic exposure + Shannon diversity**  (OR 95% CI) ^a^ | **AIC**  **R^2^** |
| --- | --- | --- | --- | --- |
| ***Core dataset*** |  |  |  |  |
| **Prior antibiotic exposure** | 7.47 (2.50, 27.94; *p*< 0.01) | 94.84  21.4 | 5.12 (1.59, 20.00; *p*< 0.01) | 89.14  31.6 |
|  |  |  |  |  |
| **Shannon diversity** |  |  | 0.46 (0.25, 0.80; *p*< 0.01) |  |
|  |  |  |  |  |
|  |  |  |  |  |
| ***UHC subset*** |  |  |  |  |
| **Prior antibiotic exposure** | 8.31 (2.20, 41.35; *p*< 0.01) | 58.12  24.8 | 5.62 (1.32, 30.16; *p*= 0.03) | 53.25  38.9 |
|  |  |  |  |  |
| **Shannon diversity** |  |  | 0.39 (0.18, 0.79; *p*= 0.01) |  |
|  |  |  |  |  |

^a^ Odds ratio (OR) (95% confidence interval (CI), *p* value)

*AIC* Akaike information criterion, *UHC* University Hospital Cologne

**Table S6e: Predictive models including prior antibiotic exposure, Shannon diversity and urinary metabo­lomic features**

| **Characteristic**  (at baseline) | **Core dataset (CD)**  (OR 95% CI) ^a,b^  AIC = 80.06 | **VIF CD** | **UHC subset (UHCS)**  (OR 95% CI) ^a,c^  AIC = 48.20 | **VIF UHCS** |
| --- | --- | --- | --- | --- |
|  |  |  |  |  |
| **Prior antibiotic exposure** | 2.57 (0.68, 10.87; *p* = 0.17) | 1.10 | 2.10 (0.32, 13.92; *p*= 0.42) | 1.20 |
|  |  |  |  |  |
| **Shannon diversity** | 0.43 (0.22, 0.80; *p*< 0.01) | 1.04 | 0.43 (0.17, 0.97; *p*= 0.049) | 1.01 |
|  |  |  |  |  |
| **Urinary NMR feature at 0.935 ppm**^d^ | 2.82 (1.47, 6.33; *p*< 0.01) | 1.08 |  |  |
|  |  |  |  |  |
| **Urinary NMR feature at 8.025 ppm**^e^ |  |  | 3.89 (1.38, 14.59; *p*= 0.02) | 1.21 |
|  |  |  |  |  |

^a^ Odds ratio (OR) (95% confidence interval (CI), *p* value)

^b^ R^2^ = 0.45

^c^ R^2^ = 0.52

^d^ Key feature identified on the basis of urinary NMR profiles in the core dataset (n = 88)

^e^ Key feature identified on the basis of urinary NMR profiles in the subset of UHC patients (n = 64)

*NMR* nuclear magnetic resonance, *ppm* parts per million, *AIC* Akaike information criterion, *VIF* variance inflation factor

**Table S6f: Predictive models including prior antibiotic exposure, *Enterococcus* dominance and urinary metabolomic features**

| **Characteristic**  (at baseline) | **UHC subset (UHCS)**  (OR 95% CI) ^a,b^  AIC = 51.72 | **VIF UHCS** |
| --- | --- | --- |
|  |  |  |
| **Prior antibiotic exposure** | 2.31 (0.38, 14.30; *p*= 0.35) | 1.22 |
|  |  |  |
| ***Enterococcus* dominance** | 1.98 (0.36, 10.61; *p*= 0.42) | 1.04 |
|  |  |  |
| **Urinary NMR feature at 8.025 ppm**^c^ | 3.94 (1.49, 13.59; *p*= 0.01) | 1.19 |
|  |  |  |

^a^ Odds ratio (OR) (95% confidence interval (CI), *p* value)

^b^ R^2^ = 0.46

^c^ Key feature identified on the basis of urinary NMR profiles in the subset of UHC patients (n = 64)

*NMR* nuclear magnetic resonance, *ppm* parts per million, *AIC* Akaike information criterion, *VIF* variance inflation factor
