# Supplementary material for: Integrating intestinal microbiome and urinary metabolome data to predict secondary infection in critically ill patients
Source: Crit Care. 2026 Mar 13;30:161. doi: 10.1186/s13054-025-05818-5 (PMC13064364; doi:10.1186/s13054-025-05818-5)
Supplement: Supplementary file 2 — Supplementary Material 2: Secondary infection characteristics. [file 13054_2025_5818_MOESM2_ESM.docx]

**Integrating intestinal microbiome and urinary metabolome data**

**to predict secondary infection in critically ill patients**

**Critical Care**

Charlotte Linz^1^, Kristiyana Tsenova^2^, Katja Dettmer^3^, Lisa Ellmann^3^, Peter J. Oefner^3^

Wolfram Gronwald^3^, Fedja Farowski^1,2^, Alina M. Rüb^1,2^, Daniel E. Freedberg^4^, Philipp Koehler^1,5,6^

Jorge Garcia Borrega^1^, Jan-Hendrik Naendrup^1^, Maria J.G.T. Vehreschild^1,2^ * and Boris Böll^1+^ *

* Contributed equally

^1^ University of Cologne, Cologne, Germany, Faculty of Medicine and University Hospital Cologne, Department I of Internal Medicine, Division of Hematology-Oncology/Critical Care Medicine/Infectious Diseases, Center for Integrated Oncology Aachen Bonn Cologne Düsseldorf (CIO ABCD)

^2^ Goethe University Frankfurt, Frankfurt am Main, Germany, University Hospital Frankfurt, Department II of Internal Medicine, Infectious Diseases

^3^ University of Regensburg, Regensburg, Germany, Institute of Functional Genomics

^4^ Columbia University, New York, United States, Division of Digestive and Liver Diseases, Mailman School of Public Health, Department of Epidemiology

^5^ University of Cologne, Cologne, Germany, Faculty of Medicine and University Hospital Cologne, Department I of Internal Medicine, Division of Clinical Immunology

^6^ University of Cologne, Cologne, Ger­many, Faculty of Medicine and University Hospital Cologne, Institute of Translational Research, Cologne Excellence Cluster on Cellular Stress Responses in Aging-Associated Diseases (CECAD)

**+** Correspondence: Boris Böll, University Hospital Cologne, Kerpener Strasse 62, Cologne, Germany, email: boris.boell@uk‑koeln.de

Additional File 1: patient enrollment, study design, and clinical characteristics of the UHC subset

**Additional File 2: secondary infection characteristics**

Additional File 3: microbiome analyses and corresponding extended findings

Additional File 4: urine analyses and corresponding extended findings

Additional File 5: classification analysis, missing data, and extended findings of the multivariable regression analysis

Additional File 6: survival analysis

**Secondary infection characteristics**

**Table S2a: Prevalence of pathogens in ICU-associated secondary infection in the core dataset**

Identifica­tion and frequency of microbial pathogens in ICU patients with secondary blood­stream infection and ventilator-associated pneumonia.

|  | **Detected pathogen** | **n/N (%) of isolates** |
| --- | --- | --- |
|  |  |  |
| **Bloodstream infection**  (15 patients with at least one episode) | | |
| Gram-positive pathogens | *Enterococcus* spp. | 10/23 (43.5%) |
|  | *Enterococcus faecium* | 5/23 |
|  | *Enterococcus faecalis* | 1/23 |
|  | Vancomycin-resistant *Enterococcus* spp. (VRE) | 4/23 |
|  | Coagulase-negative *Staphylococcus* spp. | 9/23 (39.1%) |
|  | *Staphylococcus epidermidis* | 6/32 |
|  | *Staphylococcus haemolyticus* | 2/23 |
|  | *Staphylococcus hominis* | 1/23 |
|  |  |  |
|  | *Ruminococcus gnavus* | 1/23 |
|  |  |  |
| Gram-negative pathogens  Enterobacteriaceae family | *Klebsiella pneumoniae* | 2/23 |
|  | *Enterobacter cloacae* | 1/23 |
|  |  |  |
|  |  |  |
| **Ventilator-associated pneumonia**  (14 patients) | | |
| Gram-positive pathogens | Methicillin-resistant *Staphylococcus aureus* (MRSA) | 1/10 |
|  |  |  |
| Gram-negative pathogens | *Klebsiella pneumoniae* (partly 3 MRGN^a^) | 2/10 |
| Enterobacteriaceae family | *Klebsiella oxytoca* | 1/10 |
|  | *Escherichia coli* (ESBL^b^) | 1/10 |
|  | *Proteus mirabilis* | 1/10 |
| Pseudomonadaceae family | *Pseudomonas aeruginosa* | 2/10 |
| Xanthomonadaceae family | *Stenotrophomonas maltophilia* | 1/10 |
|  |  |  |
|  |  |  |
| Pneumoviridae family | Human *metapneumovirus* | 1/10 |
|  |  |  |

^a^ 3MRGN: multidrug-resistant Gram-negative bacteria resistant to three of four relevant antibiotic groups

^b^ ESBL: extended-spectrum beta-lactamase-producing bacteria

*spp.* Species

**Table S2b: Prevalence of pathogens in ICU-associated secondary infection in the subset of UHC patients**

Identification and frequency of microbial pathogens in ICU patients with secondary bloodstream infection and ventilator-associated pneu­monia.

|  | **Detected pathogen** | **n/N (%) of isolates** |
| --- | --- | --- |
|  |  |  |
| **Bloodstream infection**  (18 patients with at least one episode) | | |
| Gram-positive pathogens | *Enterococcus* spp. | 15/32 (46.9%) |
|  | *Enterococcus faecium* | 12/32 |
|  | *Enterococcus faecalis* | 2/32 |
|  | Vancomycin-resistant *Enterococcus* spp. (VRE) | 6/32 |
|  | Coagulase-negative *Staphylococcus* spp. | 13/32 (40.6%) |
|  | *Staphylococcus epidermidis* | 6/32 |
|  | *Staphylococcus haemolyticus* | 3/32 |
|  | *Staphylococcus hominis* | 2/32 |
|  | *Staphylococcus pettenkoferi* | 1/32 |
|  | *Staphylococcus simulans* | 1/32 |
|  |  |  |
| Gram-negative pathogens  Enterobacteriaceae family | *Klebsiella pneumoniae* | 1/32 |
|  | *Enterobacter cloacae* | 1/32 |
|  | *Escherichia coli* | 1/32 |
|  |  |  |
| *Candida* spp. | *Candida albicans* | 1/32 |
|  |  |  |
|  |  |  |
| **Ventilator-associated pneumonia**  (7 patients) | | |
| Gram-negative pathogens | *Citrobacter koseri* | 1/4 |
|  | *Klebsiella pneumoniae* | 1/4 |
|  | *Escherichia coli* | 1/4 |
|  | *Pseudomonas aeruginosa* | 1/4 |
|  |  |  |

*spp.* species
