# Supplementary material for: Integrating intestinal microbiome and urinary metabolome data to predict secondary infection in critically ill patients
Source: Crit Care. 2026 Mar 13;30:161. doi: 10.1186/s13054-025-05818-5 (PMC13064364; doi:10.1186/s13054-025-05818-5)
Supplement: Supplementary file 3 — Supplementary Material 3: Microbiome analyses and corresponding extended findings. [file 13054_2025_5818_MOESM3_ESM.docx]

**Integrating intestinal microbiome and urinary metabolome data**

**to predict secondary infection in critically ill patients**

**Critical Care**

Charlotte Linz^1^, Kristiyana Tsenova^2^, Katja Dettmer^3^, Lisa Ellmann^3^, Peter J. Oefner^3^

Wolfram Gronwald^3^, Fedja Farowski^1,2^, Alina M. Rüb^1,2^, Daniel E. Freedberg^4^, Philipp Koehler^1,5,6^

Jorge Garcia Borrega^1^, Jan-Hendrik Naendrup^1^, Maria J.G.T. Vehreschild^1,2^ * and Boris Böll^1+^ *

* Contributed equally

^1^ University of Cologne, Cologne, Germany, Faculty of Medicine and University Hospital Cologne, Department I of Internal Medicine, Division of Hematology-Oncology/Critical Care Medicine/Infectious Diseases, Center for Integrated Oncology Aachen Bonn Cologne Düsseldorf (CIO ABCD)

^2^ Goethe University Frankfurt, Frankfurt am Main, Germany, University Hospital Frankfurt, Department II of Internal Medicine, Infectious Diseases

^3^ University of Regensburg, Regensburg, Germany, Institute of Functional Genomics

^4^ Columbia University, New York, United States, Division of Digestive and Liver Diseases, Mailman School of Public Health, Department of Epidemiology

^5^ University of Cologne, Cologne, Germany, Faculty of Medicine and University Hospital Cologne, Department I of Internal Medicine, Division of Clinical Immunology

^6^ University of Cologne, Cologne, Ger­many, Faculty of Medicine and University Hospital Cologne, Institute of Translational Research, Cologne Excellence Cluster on Cellular Stress Responses in Aging-Associated Diseases (CECAD)

**+** Correspondence: Boris Böll, University Hospital Cologne, Kerpener Strasse 62, Cologne, Germany, email: boris.boell@uk‑koeln.de

Additional File 1: patient enrollment, study design, and clinical characteristics of the UHC subset

Additional File 2: secondary infection characteristics

Additional File 3: microbiome analyses and corresponding extended findings

Additional File 4: urine analyses and corresponding extended findings

Additional File 5: classification analysis, missing data, and extended findings of the multivariable regression analysis

**Additional File 6: survival analysisSurvival analysis**

In the core dataset, 30 patients (34%) died. Secondary infection (SI) did not significantly impact overall survival (*p* = 0.79).

**Figure S7: Survival outcomes in the core dataset – impact of secondary infection**

Kaplan-Meier analysis


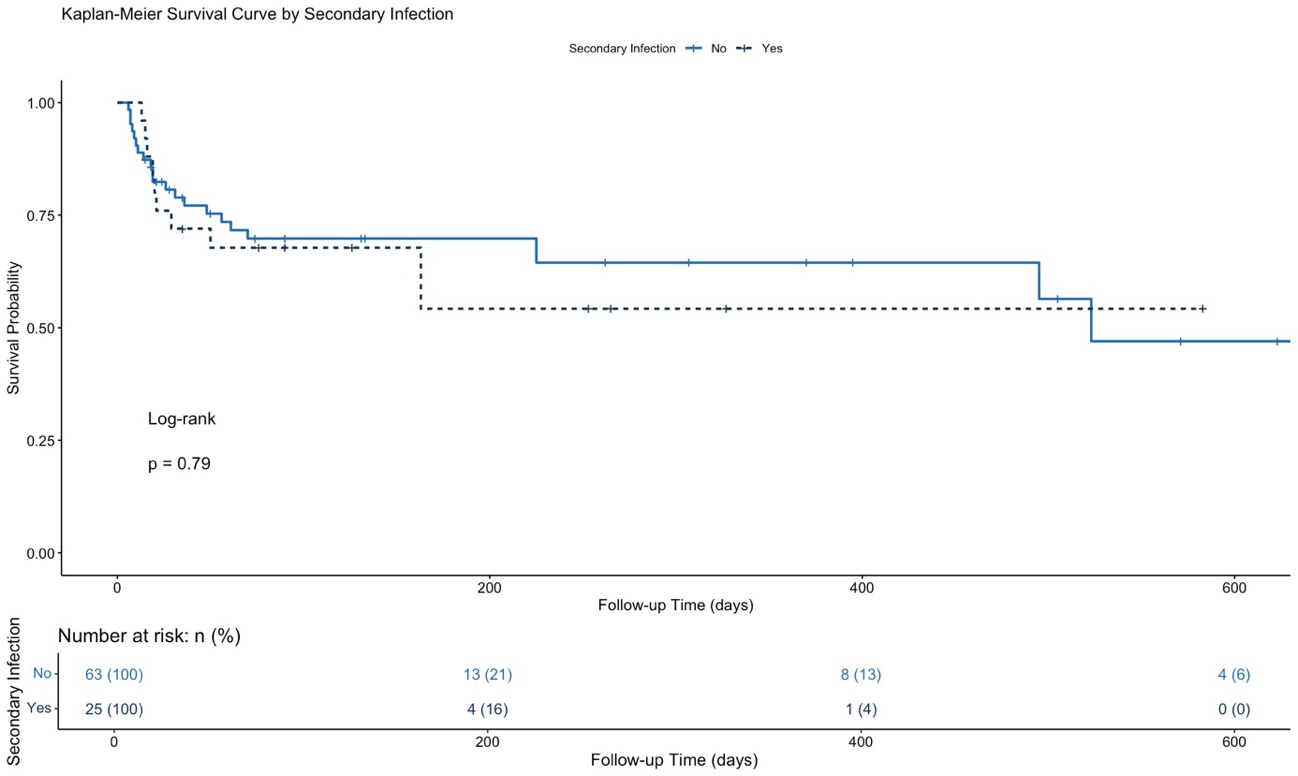


**Table S7: Comparative analysis of time-dependent survival rates in the total dataset and stratified by secondary infec­tion (SI) status.**

|  | **n** | **Total dataset**  (*n* = 88) | **Patients without SI**  (*n* = 63) | **Patients with SI**  (*n* = 25) | ***p* value**^c^ |
| --- | --- | --- | --- | --- | --- |
|  |  |  |  |  |  |
| **30-day survival**^a^ | 83 | 64 (77%) | 46 (79%) | 18 (72%) | 0.5 |
| **90-day survival**^a^ | 78 | 52 (67%) | 37 (67%) | 15 (65%) | 0.9 |
| **1-year survival**^a^ | 39 | 11 (28%) | 10 (34%) | 1 (10%) | 0.2 |
| **Overall survival**^a^ | 88 | 58 (66%) | 42 (67%) | 16 (64%) | 0.8 |
|  |  |  |  |  |  |
| **Overall survival time** [days]^b^ | 88 | 523 (225, NA^d^) | 523 (495, NA^d^) | NA^e^ (163, NA^d^) | 0.8 |
|  |  |  |  |  |  |

^a^ n (%)

^b^ Median (95% CI)

^c^ Wilcoxon rank-sum test; Fisher's exact test; log-rank test

^d^ Not estimable due to insufficient number of events.

^e^ Not estimable due to survival probability >50% or insufficient events.

In the subset of UHC patients, 34 patients (43%) died. Although SI did not significantly affect over­all survival, it was associated with a shorter median survival (67 vs. 495 days, *p* = 0.09). Mortality rates for the full subset were 72% at 30 days, 59% at 90 days, 18% at one year, and 57% overall. Patients with SI exhibited notably lower survival in both the ICU (n = 12 [52%] vs. 42 [75%], *p* = 0.048) and the hospital setting (5 [28%] vs. 32 [67%], *p* = 0.005). Additionally, SI significantly prolonged ICU stays (33 vs. 11 days, *p* < 0.001) and hospital stays (69 vs. 18 days, *p* < 0.001).

**Figure S8: Survival outcomes in the subset of UHC patients – impact of secondary infection**

Kaplan-Meier analysis


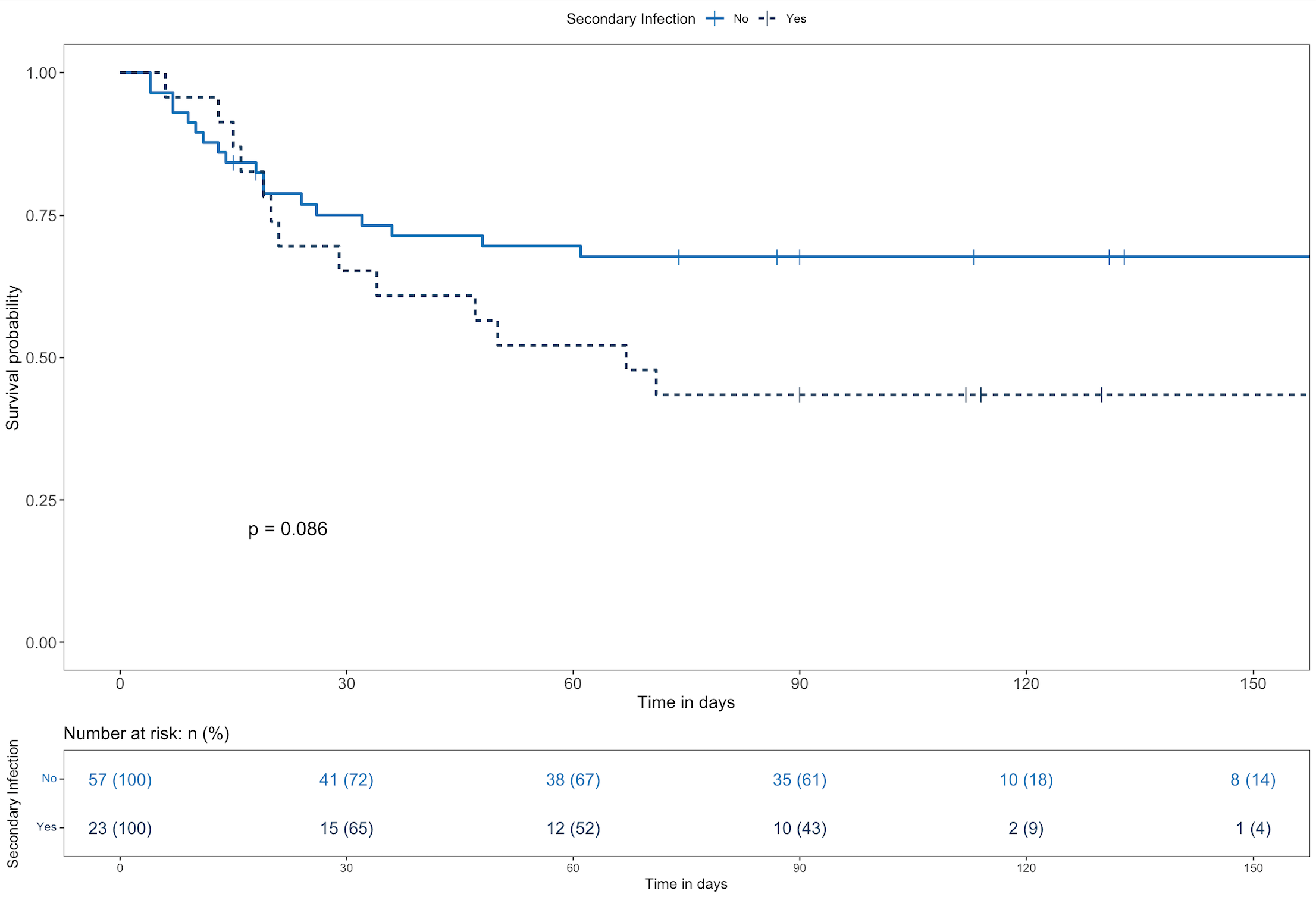


**Table S8: Comparative analysis of time-dependent and location-specific survival rates in the total subset, strati­fied by secondary infection (SI) status.**

|  | **n** | **Total subset**  (*n*= 80) | **Patients without SI**  (*n*= 57) | **Patients with SI**  (*n*= 23) | ***p* value**^d^ |
| --- | --- | --- | --- | --- | --- |
|  |  |  |  |  |  |
| **30-day survival**^a^ | 78 | 56 (72%) | 41 (75%) | 15 (65%) | 0.4 |
| **90-day survival**^a^ | 76 | 45 (59%) | 35 (66%) | 10 (43%) | 0.07 |
| **1-year survival**^a^ | 39 | 7 (18%) | 7 (27%) | 0 (0%) | 0.07 |
| **Overall survival**^a^ | 80 | 46 (58%) | 36 (63%) | 10 (43%) | 0.11 |
|  |  |  |  |  |  |
| **ICU survival**^a^ | 79 | 54 (68%) | 42 (75%) | 12 (52%) | **0.048** |
| **Hospital survival**^a^ | 66 | 37 (56%) | 32 (67%) | 5 (28%) | **< 0.01** |
| **ICU LOS** [days]^b^ | 75 | 15 (2, 71) | 11 (2, 51) | 33 (14, 71) | **< 0.01** |
| **Hospital LOS** [days]^b^ | 65 | 22 (4, 175) | 18 (4, 171) | 69 (17, 175) | **< 0.01** |
|  |  |  |  |  |  |
| **Overall survival time** [days]^c^ | 80 | 495 (225, NA^e^) | 495 (225, NA^e^) | 67 (29, NA^e^) | 0.09 |
|  |  |  |  |  |  |

^a^ n (%)

^b^ Median (range)

^c^ Median (95% CI)

^d^ Wilcoxon rank-sum test; Fisher's exact test; log-rank test

^e^ Not estimable due to insufficient number of events.

*SI* Secondary infection, *ICU* Intensive care unit, *LOS* Length of stay
