# Supplementary material for: Integrating intestinal microbiome and urinary metabolome data to predict secondary infection in critically ill patients
Source: Crit Care. 2026 Mar 13;30:161. doi: 10.1186/s13054-025-05818-5 (PMC13064364; doi:10.1186/s13054-025-05818-5)
Supplement: Supplementary file 4 — Supplementary Material 4: Urine analyses and corresponding extended findings. [file 13054_2025_5818_MOESM4_ESM.docx]

**Integrating intestinal microbiome and urinary metabolome data**

**to predict secondary infection in critically ill patients**

**Critical Care**

Charlotte Linz^1^, Kristiyana Tsenova^2^, Katja Dettmer^3^, Lisa Ellmann^3^, Peter J. Oefner^3^

Wolfram Gronwald^3^, Fedja Farowski^1,2^, Alina M. Rüb^1,2^, Daniel E. Freedberg^4^, Philipp Koehler^1,5,6^

Jorge Garcia Borrega^1^, Jan-Hendrik Naendrup^1^, Maria J.G.T. Vehreschild^1,2^ * and Boris Böll^1+^ *

* Contributed equally

^1^ University of Cologne, Cologne, Germany, Faculty of Medicine and University Hospital Cologne, Department I of Internal Medicine, Division of Hematology-Oncology/Critical Care Medicine/Infectious Diseases, Center for Integrated Oncology Aachen Bonn Cologne Düsseldorf (CIO ABCD)

^2^ Goethe University Frankfurt, Frankfurt am Main, Germany, University Hospital Frankfurt, Department II of Internal Medicine, Infectious Diseases

^3^ University of Regensburg, Regensburg, Germany, Institute of Functional Genomics

^4^ Columbia University, New York, United States, Division of Digestive and Liver Diseases, Mailman School of Public Health, Department of Epidemiology

^5^ University of Cologne, Cologne, Germany, Faculty of Medicine and University Hospital Cologne, Department I of Internal Medicine, Division of Clinical Immunology

^6^ University of Cologne, Cologne, Ger­many, Faculty of Medicine and University Hospital Cologne, Institute of Translational Research, Cologne Excellence Cluster on Cellular Stress Responses in Aging-Associated Diseases (CECAD)

**+** Correspondence: Boris Böll, University Hospital Cologne, Kerpener Strasse 62, Cologne, Germany, email: boris.boell@uk‑koeln.de

Additional File 1: patient enrollment, study design, and clinical characteristics of the UHC subset

Additional File 2: secondary infection characteristics

Additional File 3: microbiome analyses and corresponding extended findings

**Additional File 4: urine analyses and corresponding extended findings**

Additional File 5: classification analysis, missing data, and extended findings of the multivariable regression analysis

Additional File 6: survival analysis

**Urine analysis**

For NMR measurements of urine specimens, 400 μL of urine were mixed with 200 µL of 0.1 M phosphate buffer (pH 7.4), 50 µL of 0.75% (w) TSP-2,2,3,3-d4 in deuterium oxide (Sigma-Aldrich; Taufkirchen, Germany) and 10 mL of a 240 mM stock solution of formic acid, which served as an additional internal standard that, unlike TSP, does not bind to protein. Additionally, 3.9 mM of borate were added to urinary samples to prevent bacterial growth (Sigma-Aldrich; Taufkirchen, Germany). All NMR experiments were performed on a Bruker Avance III HD 600 MHz spectrometer, equipped with a Bruker SampleJet sample changer, using a triple resonance (^1^H, ^13^C, ^15^N, ^2^H lock) helium cooled cryoprobe with z-gradient (Bruker Biospin GmbH; Ettlingen, Germany). Tuning and matching of the probe as well as locking and shimming of the sample were performed automatically. 1D ^1^H NOESY spectra were acquired as described previously (1). In short, 128 scans with 64k data points were acquired with an acqui­sition and relaxation time of 2.73 and 4.0 seconds, respectively. All spectra were semiautomatically processed with TopSpin 4.14 (Bruker). For metabolite identification and quantification, the Chenomx NMR Suite 8.3 (Chenomx Inc., Edmonton, Alberta, Canada) was employed. Equidistant bucketing with a bucket width of 0.01 ppm was done using AMIX 3.9.13 (Bruker).

Urinary creatinine and 3-IS were determined by HPLC-MS/MS employing an Agilent 1200 SL HPLC and a 4000 QTRAP mass spectrometer (AB Sciex; Darmstadt, Germany). Urine specimens were either prediluted 1:5 with pure water or used directly. Ten μL of the prediluted or pure sample were mixed with 10 μL of an aqueous stable isotope-labeled internal standard mixture and 80 μL of pure water. Reversed-phase chromatographic separation was performed on an Atlantis T3 column (3 μm, 2.1x150 mm, equipped with a precolumn; Waters; Eschborn, Germany) using gradient elution with 0.1% formic acid in water as mobile phase A and 0.1% formic acid in acetonitrile as mobile phase B. The gradient started at 0% B, increased linearly to 30% B in 1 min, then to 100% over the next 8 min, was held for 1 min and returned to starting conditions with an equilibration for 6.4 min. A flow rate of 350 µL/min was used. Metabolites were detected using electrospray ionization in positive (creatinine, transition m/z 114.0 - m/z 86.0, creatinine-d3, transition m/z 117.0 - m/z 89.0) and negative mode (3-IS, transition m/z 212.0 - m/z 80, 3-IS-d4 transition m/z 216.0 - m/z 80) and multiple reaction monitoring. Calibration curves based on peak area ratio (analyte to stable isotope labeled standard) were used for quantification. 3-IS concentra­tions were normalized to creatinine.

**Metabolomic feature selection**

To address dataset heterogeneity, bucket selection was conducted based on the core dataset comprising 88 patients and 1,029 buckets, split into training (80%) and testing (20%) sets across five random seeds. Due to the dataset's high dimensionality (1029 features) and small sample size (88), a combined feature selection approach was used (2). This process generated five models, with feature selection performed using three complementary methods. First, feature importance was as­sessed using three machine learning algorithms – Random Forest (3), xgbTree (4), and glmnet (5) – across five different seeds. Features appearing in at least five sets were retained for further analysis. Second, selection by filtering based on random forest was applied, filtering features by im­portance scores based on the previous models' output (all, ≥ 5, 20, and 30), with those appearing in at least three filtered sets selected. Third, Random Forest-based Boruta was applied across 50 seeds, retaining features selected in at least 10 sets (6). Features chosen by at least two methods and appearing in at least four of five splits were included for further analysis, avoiding redundancy with duplicate occurrences counted only once. Recursive Fea­ture Elimination (RFE) was used to further refine feature selection in the UHC subset, employing 50 iterations with a Random Forest model (7).

**Results**

Metabolomic feature selection identified several NMR spectral buckets for inclusion in subsequent classification analyses, corresponding to the following metabolites. Due to the high complexity of human urine as a biofluid, substantial signal overlap is common in the resulting NMR spectra. As such, individual buckets often reflect sig­nals from multiple compounds. Where a primary contributor could be clearly identified, its name is provided; additionally, minor contributors are indicated in brackets.

***Core dataset (n = 88)***

NMR feature at 0.925 ppm: 2-oxoisocaproate and others (e.g. isoleucine)

NMR feature at 0.935 ppm: 2-oxoisocaproate and others (e.g. isoleucine)

- **2-oxoisocaproate**
  - Patients with secondary infection – median concentration: 0.004 mM/mM creatinine
  - Patients without secondary infection – median concentration: 0.002 mM/mM creatinine
- **Isoleucine**
  - Patients with secondary infection – median concentration: 0.008 mM/mM creatinine
  - Patients without secondary infection – median concentration: 0.004 mM/mM creatinine

NMR feature at 0.945 ppm: Leucine and others

NMR feature at 2.015 ppm: Several compounds

NMR feature at 2.025 ppm: Several compounds

NMR feature at 2.765 ppm: Unknown

NMR feature at 2.775 ppm: Unknown

NMR feature at 3.145 ppm: Ethanolamine

**Figure S5:** **Exemplary urine spectrum, showing the spectral region of the bucket located at 0.935 ppm**

The reference signals of isoleucine (blue) and 2-oxoisocaproate (light red) are additionally displayed.


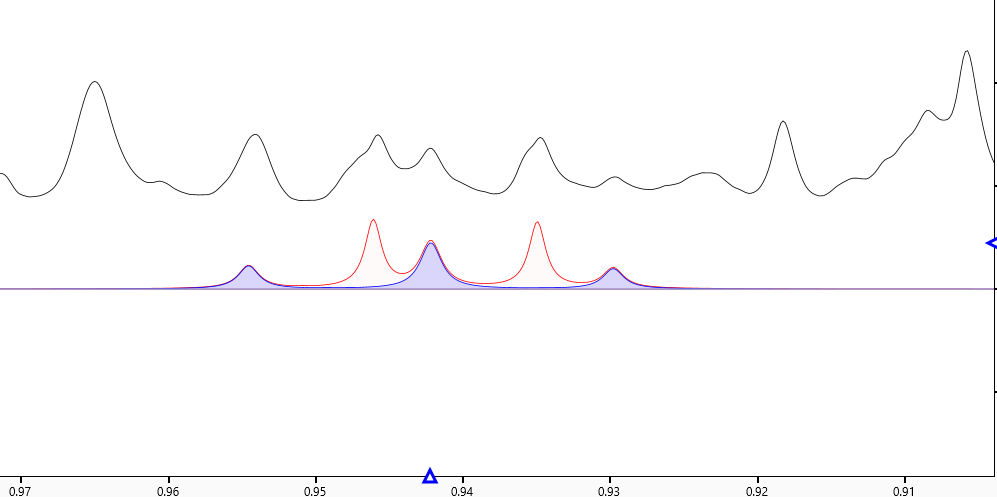


***UHC subset (n = 64)***

NMR feature at 1.029 ppm: Unknown

NMR feature at 2.015 ppm: Several compounds

NMR feature at 4.555 ppm: Multiple low-abundant compounds (e.g., carnitine)

NMR feature at 8.025 ppm: **Quinolinate**

- Patients with secondary infection – median concentration: 0.014 mM/mM creatinine
- Patients without secondary infection – median concentration: 0.011 mM/mM creatinine

**Figure S6: Association of baseline 3-indoxyl sulfate levels with subsequent secondary infection**


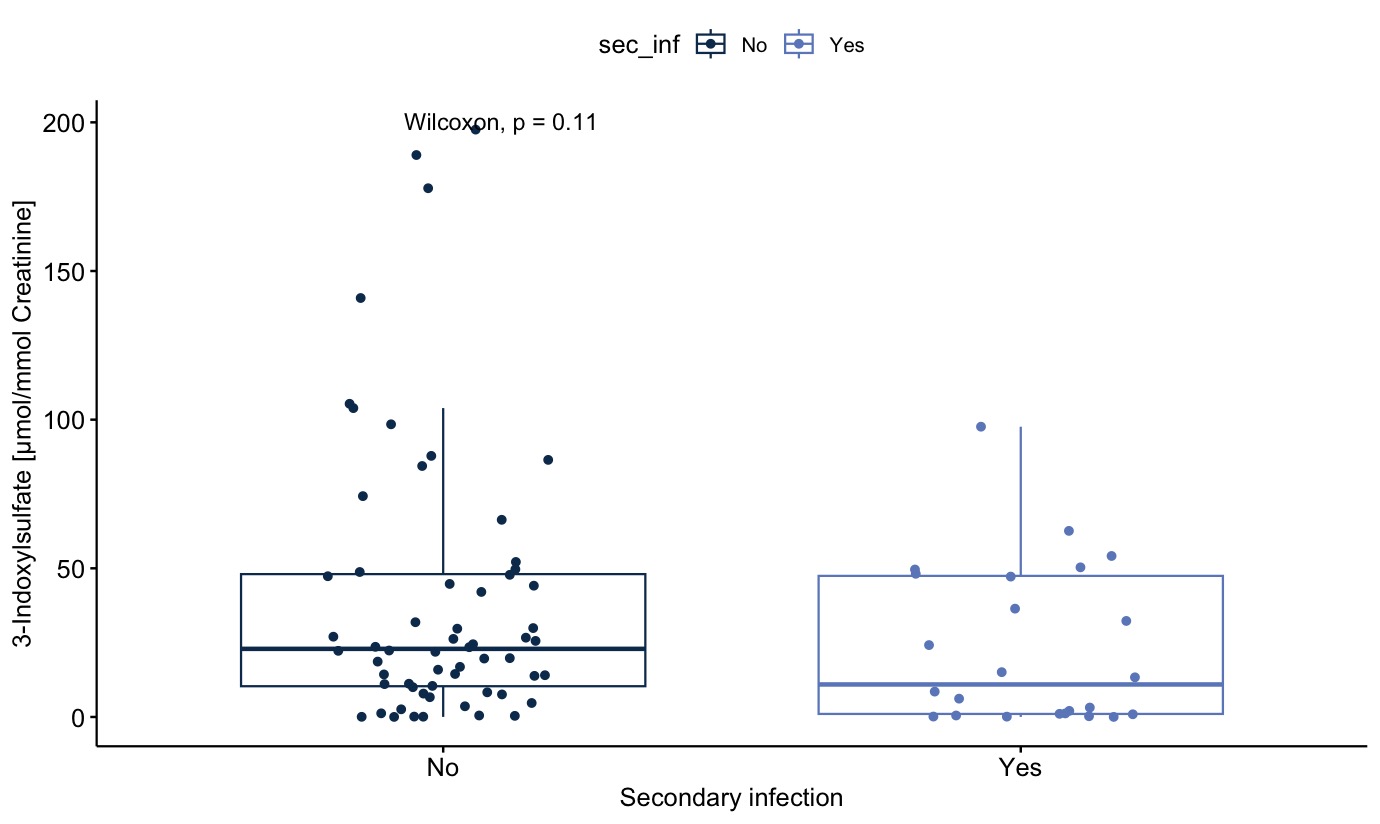


1. **Core dataset (n = 88):** Univariate analysis: Odds Ratio = 0.99, 95% Confidence Interval = 0.97, 1.00, *p* = 0.13


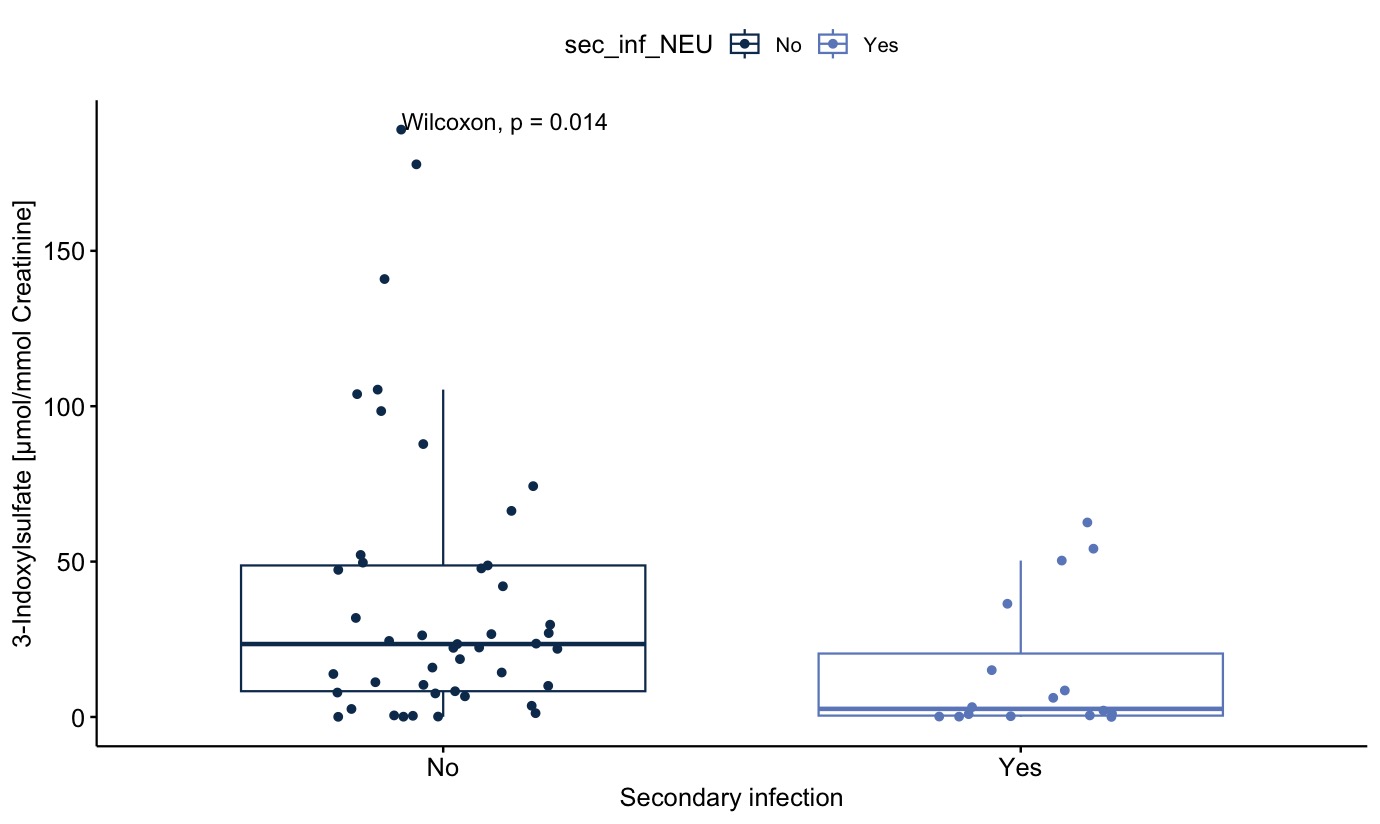


1. **UHC subset (n = 64):** Univariate analysis: Odds Ratio = 0.97, 95% Confidence Interval = 0.94, 1.00, *p* = 0.07

**References**

1. Gronwald W, Klein MS, Kaspar H, Fagerer SR, Nürnberger N, Dettmer K, et al. Urinary metabolite quantification employing 2D NMR spectroscopy. Anal Chem. 2008;80(23):9288-97.

2. Tsai C-F, Sung Y-T. Ensemble feature selection in high dimension, low sample size datasets: Parallel and serial combination approaches. Knowl Based Syst. 2020;203:106097.

3. Liaw A, Wiener, M. Classification and Regression by randomForest. R News. 2002;2(3):18-22.

4. Chen T, He T, Benesty M, Khotilovich V, Tang Y, Cho H, Chen K, Mitchell R, Cano I, Zhou T, Li M, Xie J, Lin M, Geng Y, Li Y, Yuan J (2024). xgboost: Extreme Gradient Boosting. R package version 1.7.8.1, <https://CRAN.R-project.org/package=xgboost>.

5. Friedman J, Tibshirani R, Hastie T. Regularization Paths for Generalized Linear Models via Coordinate Descent. J Stat Softw. 2010;33(1):1-22.

6. Kursa MB, Rudnicki WR. Feature Selection with the Boruta Package. J Stat Softw. 2010;36(11):1-13.

7. Kuhn M. Building Predictive Models in R Using the caret Package. J Stat Softw. 2008;28(5):1–26.
