# Supplementary material for: Integrating intestinal microbiome and urinary metabolome data to predict secondary infection in critically ill patients
Source: Crit Care. 2026 Mar 13;30:161. doi: 10.1186/s13054-025-05818-5 (PMC13064364; doi:10.1186/s13054-025-05818-5)
Supplement: Supplementary file 5 — Supplementary Material 5: Classification analysis, missing data, and extended findings of the multivariable regression analysis. [file 13054_2025_5818_MOESM5_ESM.docx]

**Integrating intestinal microbiome and urinary metabolome data**

**to predict secondary infection in critically ill patients**

**Critical Care**

Charlotte Linz^1^, Kristiyana Tsenova^2^, Katja Dettmer^3^, Lisa Ellmann^3^, Peter J. Oefner^3^

Wolfram Gronwald^3^, Fedja Farowski^1,2^, Alina M. Rüb^1,2^, Daniel E. Freedberg^4^, Philipp Koehler^1,5,6^

Jorge Garcia Borrega^1^, Jan-Hendrik Naendrup^1^, Maria J.G.T. Vehreschild^1,2^ * and Boris Böll^1+^ *

* Contributed equally

^1^ University of Cologne, Cologne, Germany, Faculty of Medicine and University Hospital Cologne, Department I of Internal Medicine, Division of Hematology-Oncology/Critical Care Medicine/Infectious Diseases, Center for Integrated Oncology Aachen Bonn Cologne Düsseldorf (CIO ABCD)

^2^ Goethe University Frankfurt, Frankfurt am Main, Germany, University Hospital Frankfurt, Department II of Internal Medicine, Infectious Diseases

^3^ University of Regensburg, Regensburg, Germany, Institute of Functional Genomics

^4^ Columbia University, New York, United States, Division of Digestive and Liver Diseases, Mailman School of Public Health, Department of Epidemiology

^5^ University of Cologne, Cologne, Germany, Faculty of Medicine and University Hospital Cologne, Department I of Internal Medicine, Division of Clinical Immunology

^6^ University of Cologne, Cologne, Ger­many, Faculty of Medicine and University Hospital Cologne, Institute of Translational Research, Cologne Excellence Cluster on Cellular Stress Responses in Aging-Associated Diseases (CECAD)

**+** Correspondence: Boris Böll, University Hospital Cologne, Kerpener Strasse 62, Cologne, Germany, email: boris.boell@uk‑koeln.de

**Additional File 1: patient enrollment, study design, and clinical characteristics of the UHC subset**

Additional File 2: secondary infection characteristics

Additional File 3: microbiome analyses and corresponding extended findings

Additional File 4: urine analyses and corresponding extended findings

Additional File 5: classification analysis, missing data, and extended findings of the multivariable regression analysis

Additional File 6: survival analysis

**Patient enrollment**

Our core dataset comprised 88 patients from two independent academic medical centers: University Hospital Cologne (UHC), Germany (n = 64), enrolled between January 2019 – July 2021, and Columbia University Medical Center (CUMC), New York, United States of America (n = 24), enrolled between February 2015 – August 2018. Urine samples collected at admission were available for all core dataset patients. To investigate microbiome signatures in greater detail with more comprehensive data available, we performed additional analyses on a subset of UHC patients (n = 80), which comprised the original 64 core patients plus 16 additional UHC patients (enrolled between January 2019 – July 2021). This subset provided more comprehensive microbiome profiling data, including extended characterization of the intestinal microbiome, as well as bronchoalveolar lavage fluid and endotracheal aspirate samples, allowing deeper taxonomic characterization and evaluation of the respiratory tract microbiome. Within this expanded clinical cohort of 80 patients, 69 patients had microbiome data available, 64 patients had urinary metabolome data available and 55 individuals had complete microbiome and urinary metabolome data available, forming the dataset used for integrated predictive modeling.

**Figure S1: Patient enrollment and data availability**

This figure illustrates the composition of the core dataset, which includes patients from Columbia University Medical Center (CUMC) and University Hospital Cologne (UHC), alongside the UHC subset. To accommodate the differing focuses of the two analytical phases, imputation was applied selectively: for the core dataset, missing clinical and microbiome data (specifically, Shannon diversity missing in 9 of 88 patients) were imputed, resulting in a complete dataset of n = 88. In contrast, for the UHC subset, only missing clinical data were imputed, while microbiome and metabolome data with missing values were excluded, yielding a fully complete dataset of n = 55. Additional information on imputation is available in Supplementary Table S5, Additional File 5.


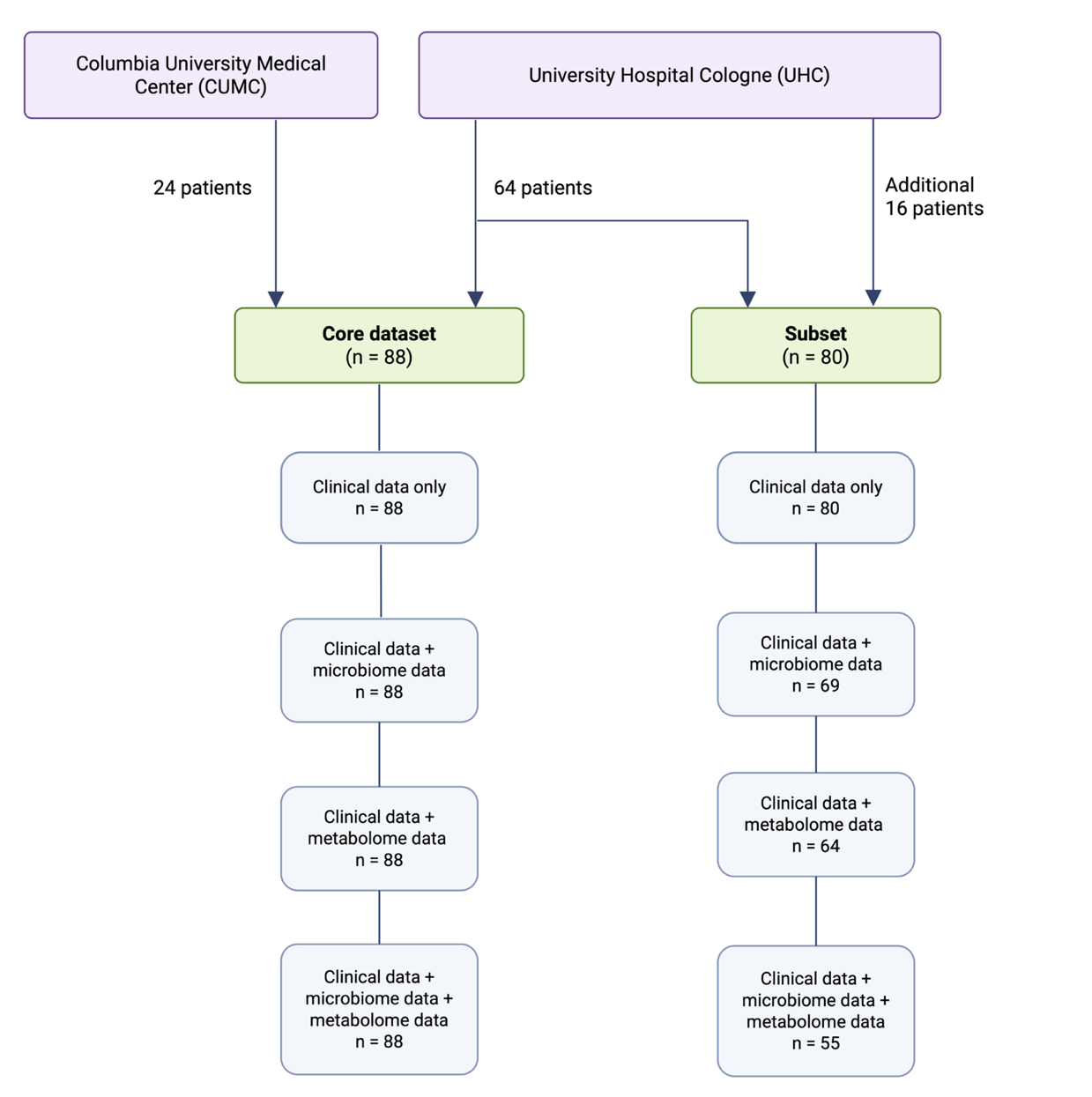


**Data collection and clinical outcome definition**

For the UHC cohort, clinical and laboratory values were recorded as the median value on the day of sample col­lection when multiple measurements were available (e.g., vital signs or blood gas parameters); most laboratory values were captures as a single daily value. All secondary infection events occurring within the 20-day observa­tional period were considered. In the CUMC cohort, clinical and laboratory values instead corresponded to the worst value documented within the 24 hours preceding each sample collection. Secondary infection events were included if they occurred within the 30-day follow-up period.

**Patient characteristics (UHC subset)**

**Table S1:** **Patient characteristics**

Demographic and clinical characteristics of patients included in the extended subset of UHC patients (n = 80). Comparative summary of the overall subset and subgroups stratified by secondary infection (SI) status.

|  | **Total subset**  (*n*= 80)  n (%), median (IQR) | **Patients without SI**  (*n*= 57)  n (%), median (IQR) | **Patients with SI**  (*n*= 23)  n (%), median (IQR) | ***q* value**^a^ |
| --- | --- | --- | --- | --- |
|  |  |  |  |  |
| **Age** (years) | 59 (49, 69) | 62 (52, 71) | 55 (46, 62) | 0.13 |
| **Male Gender** | 46 (58%) | 33 (58%) | 13 (57%) | > 0.99 |
| **Karnofsky Performance Status Scale** | 60 (50, 80) | 60 (50, 80) | 70 (43, 80) | > 0.99 |
|  |  |  |  |  |
| **ICU admission characteristics** | | | |  |
| ***Origin*** |  |  |  | **< 0.01** |
| In-house hospital ward | 32 (40%) | 14 (25%) | 18 (78%) |  |
| Emergency department | 31 (39%) | 30 (53%) | 1 (4.3%) |  |
| External hospital | 17 (21%) | 13 (23%) | 4 (17%) |  |
|  |  |  |  |  |
| ***Admission diagnosis***^b^ |  |  |  |  |
| Respiratory failure | 55 (69%) | 38 (67%) | 17 (74%) | 0.91 |
| Sepsis | 49 (61%) | 34 (60%) | 15 (65%) | > 0.99 |
| Shock | 30 (38%) | 21 (37%) | 9 (39%) | > 0.99 |
| Neurological condition | 14 (18%) | 9 (16%) | 5 (22%) | 0.91 |
| Cardiovascular condition | 13 (16%) | 12 (21%) | 1 (4.3%) | 0.32 |
| Other | 14 (18%) | 10 (18%) | 4 (17%) | > 0.99 |
|  |  |  |  |  |
| **Comorbidities** |  |  |  |  |
| Cardiovascular | 53 (66%) | 36 (63%) | 17 (74%) | 0.82 |
| Hemato-oncological | 47 (59%) | 28 (49%) | 19 (83%) | **0.045** |
| Gastrointestinal | 39 (49%) | 27 (47%) | 12 (52%) | > 0.99 |
| Endocrine | 38 (48%) | 27 (47%) | 11 (48%) | > 0.99 |
| Pulmonary | 33 (41%) | 22 (39%) | 11 (48%) | 0.90 |
| Neurological | 30 (38%) | 23 (40%) | 7 (30%) | 0.88 |
| Renal | 23 (29%) | 17 (30%) | 6 (26%) | > 0.99 |
| Orthopedic | 15 (19%) | 10 (18%) | 5 (22%) | > 0.99 |
| Urological | 14 (18%) | 10 (18%) | 4 (17%) | > 0.99 |
| Psychiatric | 12 (15%) | 10 (18%) | 2 (8.7%) | 0.90 |
| Rheumatic | 3 (3.8%) | 2 (3.5%) | 1 (4.3%) | > 0.99 |
| No comorbidities | 1 (1.3%) | 1 (1.8%) | 0 (0%) | > 0.99 |
| **CCI** | 4 (2, 7) | 5 (3, 7) | 4 (2, 5) | 0.39 |
| **Allogeneic stem cell trans­plant** | 13 (16%) | 6 (11%) | 7 (30%) | 0.19 |
| **Organ transplantation** | 2 (2.5%) | 1 (1.8%) | 1 (4.3%) | 0.90 |
| **Chronic dialysis** | 4 (5.0%) | 2 (3.5%) | 2 (8.7%) | 0.93 |
|  |  |  |  |  |
| **Medication preceding ICU admission** | | | | |
| Antibiotic treatment^c^ | 37 (46%) | 19 (33%) | 18 (78%) | **< 0.01** |
| Immunosuppressive therapy^d^ | 20 (25%) | 9 (16%) | 11 (48%) | **0.02** |
| Chemotherapy^d^ | 19 (24%) | 8 (14%) | 11 (48%) | **0.01** |
| Immunotherapy^e^ | 7 (8.8%) | 2 (3.5%) | 5 (22%) | 0.11 |
|  |  |  |  |  |
| **Measures of disease severity and mortality prediction at baseline** | | | | |
| qSOFA |  |  |  | 0.88 |
| 1 | 2 (2.6%) | 1 (1.8%) | 1 (5.0%) |  |
| 2 | 37 (49%) | 30 (54%) | 7 (35%) |  |
| 3 | 25 (33%) | 17 (30%) | 8 (40%) |  |
| 4 | 12 (16%) | 8 (14%) | 4 (20%) |  |
| APACHE II | 25 (19, 31) | 23 (18, 30) | 29 (25, 33) | 0.13 |
| Septic shock | 26 (33%) | 17 (30%) | 9 (39%) | 0.87 |
| SIRS ≥ 2 | 44 (59%) | 30 (55%) | 14 (74%) | 0.39 |
| Acute renal failure | 32 (40%) | 20 (35%) | 12 (52%) | 0.42 |
| Core-10-TISS | 15 (10, 18) | 15 (10, 18) | 15 (12, 18) | > 0.99 |
| SAPS II | 42 (31, 51) | 40 (29, 50) | 46 (35, 54) | 0.32 |
|  |  |  |  |  |
| **Mechanical ventilation parameters at baseline** | | | | |
| Tidal volume (ml) | 410 (357, 468) | 417 (369, 468) | 395 (296, 470) | 0.57 |
| PEEP (mbar) | 7.1 (6, 9) | 7.4 (6, 9) | 7.1 (6, 8.45) | > 0.99 |
| p_a_O_2_ [mmHg] | 92 (76, 113) | 93 (76, 111) | 88 (74, 114) | > 0.99 |
| p_a_CO_2_ [mmHg] | 47 (39, 55) | 48 (39, 56) | 45 (40, 53) | > 0.99 |
| F_i_O_2_ [%] | 39 (33, 50) | 42 (33, 52) | 38 (35, 48) | > 0.99 |
| p_a_O_2_/F_i_O_2_ | 228 (155, 295) | 225 (149, 276) | 251 (180, 308) | 0.39 |
|  |  |  |  |  |
| **Clinical and laboratory data at baseline** | | | | |
| WBC [x 10^9^/L] | 11 (6, 16) | 12 (7, 17) | 10 (1, 15) | 0.33 |
| Platelet count  [x 10^9^/L] | 150 (67, 274) | 180 (86, 294) | 75 (26, 163) | **0.046** |
| Hematocrit [%] | 29 (24, 34) | 31 (26, 38) | 23 (21, 28) | **< 0.01** |
| Hemoglobin [g/dL] | 9.85 (8.60, 12.50) | 11.10 (8.80, 13.00) | 8.70 (7.95, 9.95) | **< 0.01** |
| Creatinine [mg/dL] | 1.14 (0.79, 2.25) | 1.07 (0.79, 1.72) | 1.35 (0.81, 2.59) | > 0.99 |
| Bilirubin [mg/dL] | 0.7 (0.3, 1.4) | 0.6 (0.3, 1.2) | 0.9 (0.4, 2.0) | 0.39 |
| CRP [mg/L] | 51 (13, 127) | 35 (8, 122) | 68 (31, 153) | 0.32 |
| PCT [µg/L] | 1 (1, 4) | 1 (0, 6) | 1 (1, 3) | 0.92 |
| Min. pH | 7.2 (7.2, 7.3) | 7.3 (7.2, 7.3) | 7.2 (7.1, 7.3) | 0.73 |
|  |  |  |  |  |
| Max. temperature [°C] | 37.6 (36.8, 38.2) | 37.5 (36.8, 38.2) | 37.9 (36.4, 38.2) | > 0.99 |
| MAP [mmHg] | 75 (70, 83) | 75 (70, 84) | 75 (70, 80) | > 0.99 |
| Max. heart rate [bpm] | 109 (90, 140) | 109 (90, 141) | 106 (91, 134) | > 0.99 |
| Max. respiratory rate [bpm] | 23 (18, 32) | 22 (18, 28) | 30 (19, 40) | 0.32 |
|  |  |  |  |  |

^a^ Wilcoxon rank sum test, Pearson’s Chi-squared test, Fisher’s exact test; false discovery rate correction for multiple testing

^b^ This variable allowed for multiple answers.

^c^ Within six months preceding ICU admission, including treatment at the time of admission, coded as a binary variable (yes/no), encompassing both broad- and narrow-spectrum antibiotics

^d^ Three months preceding ICU admission

^e^ Three months preceding ICU admission; includes checkpoint inhibitors, antibody therapy, CAR-T cell therapy and other immunomodulatory treatments

*IQR* interquartile range, *SI* secondary infection, *BMI* body mass index, *CCI* Charlson Comorbidity Index, *ICU* intensive care unit, *qSOFA* quick Sequential Organ Failure Assessment, *APACHE II* Acute Physiology-Age-Chronic Health Evaluation II, *SIRS* Systemic Inflammatory Response Syndrome, *TISS* Therapeutic Intervention Scoring System, *SAPS II* Simplified Acute Physiology Score II, *PEEP* positive end-expiratory pressure, *WBC* white blood cell count, *CRP* C-reactive protein, *PCT* procalcitonin*, MAP* mean arterial pressure
