# Supplementary material for: Integrating intestinal microbiome and urinary metabolome data to predict secondary infection in critically ill patients
Source: Crit Care. 2026 Mar 13;30:161. doi: 10.1186/s13054-025-05818-5 (PMC13064364; doi:10.1186/s13054-025-05818-5)
Supplement: Supplementary file 6 — Supplementary Material 6: Survival analysis. [file 13054_2025_5818_MOESM6_ESM.docx]

**Integrating intestinal microbiome and urinary metabolome data**

**to predict secondary infection in critically ill patients**

**Critical Care**

Charlotte Linz^1^, Kristiyana Tsenova^2^, Katja Dettmer^3^, Lisa Ellmann^3^, Peter J. Oefner^3^

Wolfram Gronwald^3^, Fedja Farowski^1,2^, Alina M. Rüb^1,2^, Daniel E. Freedberg^4^, Philipp Koehler^1,5,6^

Jorge Garcia Borrega^1^, Jan-Hendrik Naendrup^1^, Maria J.G.T. Vehreschild^1,2^ * and Boris Böll^1+^ *

* Contributed equally

^1^ University of Cologne, Cologne, Germany, Faculty of Medicine and University Hospital Cologne, Department I of Internal Medicine, Division of Hematology-Oncology/Critical Care Medicine/Infectious Diseases, Center for Integrated Oncology Aachen Bonn Cologne Düsseldorf (CIO ABCD)

^2^ Goethe University Frankfurt, Frankfurt am Main, Germany, University Hospital Frankfurt, Department II of Internal Medicine, Infectious Diseases

^3^ University of Regensburg, Regensburg, Germany, Institute of Functional Genomics

^4^ Columbia University, New York, United States, Division of Digestive and Liver Diseases, Mailman School of Public Health, Department of Epidemiology

^5^ University of Cologne, Cologne, Germany, Faculty of Medicine and University Hospital Cologne, Department I of Internal Medicine, Division of Clinical Immunology

^6^ University of Cologne, Cologne, Ger­many, Faculty of Medicine and University Hospital Cologne, Institute of Translational Research, Cologne Excellence Cluster on Cellular Stress Responses in Aging-Associated Diseases (CECAD)

**+** Correspondence: Boris Böll, University Hospital Cologne, Kerpener Strasse 62, Cologne, Germany, email: boris.boell@uk‑koeln.de

Additional File 1: patient enrollment, study design, and clinical characteristics of the UHC subset

Additional File 2: secondary infection characteristics

**Additional File 3: microbiome analyses and corresponding extended findings**

Additional File 4: urine analyses and corresponding extended findings

Additional File 5: classification analysis, missing data, and extended findings of the multivariable regression analysis

Additional File 6: survival analysis**Microbiome analysis**

Note: microbiome analyses in the core dataset (UHC and CUMC patients) were limited to the assessment of intestinal Shannon diversity. Extended analyses were subsequently conducted in an expanded UHC subset, encompassing the 64 UHC patients from the core dataset together with 16 additional UHC patients.

Microbiome analysis for the CUMC cohort followed the methodology described by Kuo et al. (2018) (1).

For the UHC cohort, microbiome analysis was performed as follows:

**Specimen collection and initial processing (within 48 hours of intubation)**

- Fecal swabs (collected by rotating the swab in stool) and rectal swabs: collected using a COPAN eSwab™ and placed in a transport tube containing 3.5 mL of ZymoBIOMICS™ DNA/RNA Shield
- BAL/ETA: 2.5 mL of BAL or tracheal secretion fluid were collected in a transport tube containing 2.5 mL of ZymoBIOMICS™ DNA/RNA Shield
- Urine specimens: 8 mL were collected via the collection port of an indwelling catheter
- All specimens, including swabs when applicable, were stored at −80 °C in ZymoBIOMICS™ DNA/RNA Shield (Zymo Research; Irvine, CA, USA) within 24 hours of collection for subsequent batched pro­cessing.

**DNA extraction protocol**

DNA was extracted using the ZymoBIOMICS™ DNA/RNA Miniprep Kit according to the manufacturer’s in­structions, with minor modifications. Although RNA was co-extracted as part of the broader project framework and is referenced accordingly, it was not utilized further in the present study. Quantification and purity assessment of nucleic acids were performed using a NanoDrop™ One Microvolume UV-Vis Spectrophotometer (Thermo Scientific; Waltham, MA, USA) following the manufacturer’s guidelines.

**Sequencing protocol**

16S rRNA amplicon sequencing targeting the V3 – V4 hypervariable regions was performed according to the ‘16S Metagenomic Sequencing Library Preparation Guide’ for the ‘Illumina MiSeq System’ (Illumina; San Diego, CA, USA) (https://support.illumina.com/content/dam/illumina-sup­port/documents/documentation/chemistry_documentation/16s/16s-metagenomic-library-prep-guide-15044223-b.pdf). DNA quantification was conducted using the Qubit™ dsDNA High Sensitivity (HS) Assay (Thermo Sci­entific; Waltham, MA, USA), a fluorometric method for double-stranded DNA measurement. Samples were di­luted to a concentration of 5 ng/µL based on Qubit™ results.

The 16S rRNA amplicon PCR setup included microbial DNA, diluted J12 forward and J13 reverse primers, and KAPA HiFi HotStart ReadyMix. Primers were dispensed into each well of a microtiter plate, followed by sample addition and mixing with the reaction mix. The sealed plate was briefly shaken and centrifuged before thermocy­cling, which consisted of an initial denaturation at 95 °C, followed by 25 cycles of denaturation (95 °C), annealing (55 °C), and extension (72 °C), with a final extension at 72 °C and a hold at 4 °C. For low-biomass samples (< 5 ng/μL), the input volume was increased by reducing the amount of water in the primer mix, effectively allow­ing more sample to be used. All subsequent steps followed the standard protocol.

Amplicon PCR clean-up was performed using AMPure XP magnetic beads (Beckman Coulter; Brea, CA, USA) to remove unincorporated primers, nucleotides, and other contaminants. Beads were brought to room temperature, centrifuged, vortexed, and added to each well. After incubation, plates were placed on a magnetic stand to allow clearance of the supernatant, followed by two washes with 80% ethanol, air drying, and elution with elution buffer. The eluate was transferred to a new PCR plate.

For index PCR, the KAPA HiFi HotStart ReadyMix (Roche Sequencing; Pleasanton, CA, USA) and Illumina UD Index Primer Plate were thawed, and a reaction mix (KAPA Hifi and PCR water) was prepared. UD Index Primer solutions were mixed and centrifuged before being transferred to a new microwell plate, followed by the addition of the reaction mix. The 16S amplicon product was then added, and the plate sealed, briefly shaken, and centri­fuged. Thermocycling followed a reduced protocol of 8 cycles. Index PCR clean-up was conducted similarly to the amplicon PCR clean-up, with minor adjustments in reagent volumes and concentrations, as outlined in the protocol.

PCR efficiency was verified using the Qiagen QIAxcel System (Qiagen; Hilden, Germany), a capillary electro­phoresis platform. Elution buffer, standard size markers, and diluted samples were dispensed into microwell plates, sealed, centrifuged, and analyzed.

Library quantification was performed using the Qubit™ dsDNA HS Assay to determine DNA concentrations for calculating molar equivalents. Equimolar concentrations of individual libraries were then pooled to ensure bal­anced sequencing depth across samples. For sequencing preparation, pooled libraries were denatured with NaOH, incubated, and diluted with pre-chilled hybridization buffer. A PhiX control library (PhiX Control v3; Illumina; San Diego, CA, USA) was prepared in parallel, denatured, and diluted to match the amplicon library concentration, ensuring a minimum of 5% PhiX content. The denatured libraries were combined on ice and heat-denatured im­mediately before loading onto the MiSeq v3 reagent cartridge. Sequencing was conducted on the Illumina MiSeq platform.

**Processing of Sequencing Data**

Sequencing data were processed using the DADA2 pipeline (2) and QIIME2 (3). Reads were trimmed and pro­cessed by the QIIME2 DADA2 plugin with the denoise-paired option and standard parameters (trunc_q = 2, max_ee = 2, chimera_method = consensus). Taxonomic classifications were assigned via a Naïve Bayes classifier (scikit-learn), trained on the SILVA database release 138. Microbiota analyses were carried out using R for Statistical Computing (version 4.2.0, R Foundation for Statistical Computing, Vienna, Austria). The QIIME BIOM data was imported and diversity scores calculated using the phyloseq R package (4).

**Microbial diversity and differential abundance analysis**

Microbial domination was established as the presence of a single taxon representing ≥ 30% of the relative abun­dance in a given sample, which served as the threshold for identifying taxa with overrepresentation. Alpha diversity (α-diversity) was assessed using the Shannon and Simpson's diversity index, the Abundance-based Cover­age Estimator (ACE), Chao1, species richness (SR), and phylogenetic diversity (PD). Beta diversity (β-diversity) was evaluated using generalized, weighted and unweighted UniFrac metrics, along with Bray-Curtis dissimilarity. Distance matrices were visualized via principal coordinate analysis (PCoA), and group differences were tested with permutational multivariate analysis of variance (PERMANOVA) (5).

Differential abundance analysis was performed using linear discriminant analysis (LDA) effect size (LEfSe). Taxa with an LDA score > 2 at p < 0.05 (determined by Kruskal–Wallis test) were considered significantly en­riched. LEfSe analysis was conducted locally using the LEfSe tool.

**Microbiome analysis results**

(UHC subset)

**Intestinal microbiome**

**Table S3a: Intestinal microbiome: relative abundance of top taxa in the intestinal microbiome**

| **Taxa** | **Full patient set**^a^ | **Patients without SI**^b^ | **Patients with SI**^b^ | ***q* value**^c^ |
| --- | --- | --- | --- | --- |
|  |  |  |  |  |
| **Class level** |  |  |  |  |
| Bacilli | 39.6 (0.5 – 100.0) | 18.7 | 60.5 | **< 0.01** |
| Clostridia | 27.9 (0 – 77.7) | 36.8 | 19.0 | **0.04** |
| Bacteroidia | 13.2 (0 – 41.5) | 19.9 | 6.5 | **< 0.01** |
| Gammaproteobacteria | 7.9 (0 – 92.9) | 11.6 | 4.1 | **0.01** |
|  |  |  |  |  |
| **Genus level** |  |  |  |  |
| *Enterococcus* | 28.8 (0 – 100.0) | 11.2 | 46.3 | **< 0.01** |
| *Bacteroides* | 7.1 (0 – 34.3) | 10.9 | 3.3 | **0.02** |
| *Finegoldia* | 4.9 (0 – 43.2) | 5.9 | 4.0 | 0.13 |
|  |  |  |  |  |

^a^ Mean (min – max) [%]

^b^ Mean [%]

^c^ Wilcoxon rank-sum test; false discovery rate correction for multiple testing

*SI* Secondary infection

**Table S3b: Intestinal microbiome: frequency of dominance (relative abundance ≥ 30%) among the most frequently dominant phyla in the intestinal microbiome**

| **Taxa at phylum level** | **Full patient set**^a^ | **Patients without SI**^a^ | **Patients with SI**^a^ | ***q* value**^b^ |
| --- | --- | --- | --- | --- |
|  |  |  |  |  |
| Actinobacteriota | 3 (4.3%) | 2 (4.1%) | 1 (5.0%) | > 0.9 |
| Bacteroidota | 3 (4.3%) | 2 (4.1%) | 1 (5.0%) | > 0.9 |
| Firmicutes | 25 (36%) | 11 (22%) | 14 (70%) | **< 0.01** |
| Proteobacteria | 3 (4.3%) | 2 (4.1%) | 1 (5.0%) | > 0.9 |
|  |  |  |  |  |

^a^ n (%)

^b^ Fisher's exact test; Pearson's Chi-squared test; false discovery rate correction for multiple testing

*SI* Secondary infection

**Table S3c: Intestinal microbiome: α-diversity of the intestinal microbiome**

|  | **n** | **Full patient set**^a^ | **Patients without SI**^a^ | **Patients with SI**^a^ | ***p* value**^b^ |
| --- | --- | --- | --- | --- | --- |
|  |  |  |  |  |  |
| Shannon | 69 | 3.18 (1.71, 3.90) | 3.65 (2.71, 4.11) | 1.55 (1.02, 2.98) | **< 0.01** |
| Simpson | 69 | 0.92 (0.72, 0.96) | 0.95 (0.89, 0.97) | 0.69 (0.45, 0.91) | **< 0.01** |
| ACE | 64 | 133 (47, 182) | 156 (109, 188) | 46 (18, 83) | **< 0.01** |
| Chao1^c^ | 69 | 116 (45, 180) | 155 (61, 187) | 44 (18, 65) | **< 0.01** |
| PD | 69 | 7.1 (3.1, 9.3) | 8.4 (5.0, 9.7) | 3.3 (1.3, 5.7) | **< 0.01** |
| SR^c^ | 69 | 116 (45, 180) | 155 (61, 187) | 44 (18, 65) | **< 0.01** |
|  |  |  |  |  |  |

^a^ Median (IQR)

^b^ Wilcoxon rank-sum test; Wilcoxon rank-sum exact test

^c^ Chao1 and SR values were identical across all samples due to the absence of singletons.

*SI* Secondary infection, *ACE* Abundance-based coverage estimator, *PD* Phylogenetic Diversity, *SR* Species rich­ness

**Figure S2a:** **Principal coordinate analysis (PCoA)** based on generalized (left) and unweighted UniFrac (right) dis­similarity met­rics of intestinal microbiome data. Solid ellipses indicate the 95% confidence intervals of the group centroids, while dashed ellipses represent the area expected to contain 95% of the data points for each group, assuming a multivariate normal distribution. The percentage of variance explained by each axis is shown.

Generalized UniFrac: R^2^ = 0.07, F = 5.19, *p* < 0.01

Unweighted UniFrac: R^2^ = 0.06, F = 4.34, *p* < 0.01

Bray-Curtis dissimilarity: R^2^ = 0.05, F = 3.74, *p* < 0.01

**Figure S2b: Linear discriminant analysis (LDA) effect size (LEfSe) analysis**

The histogram details the LDA score, with bar lengths indicating the LDA score's magnitude, reflecting how strongly each taxon differentiates between groups.


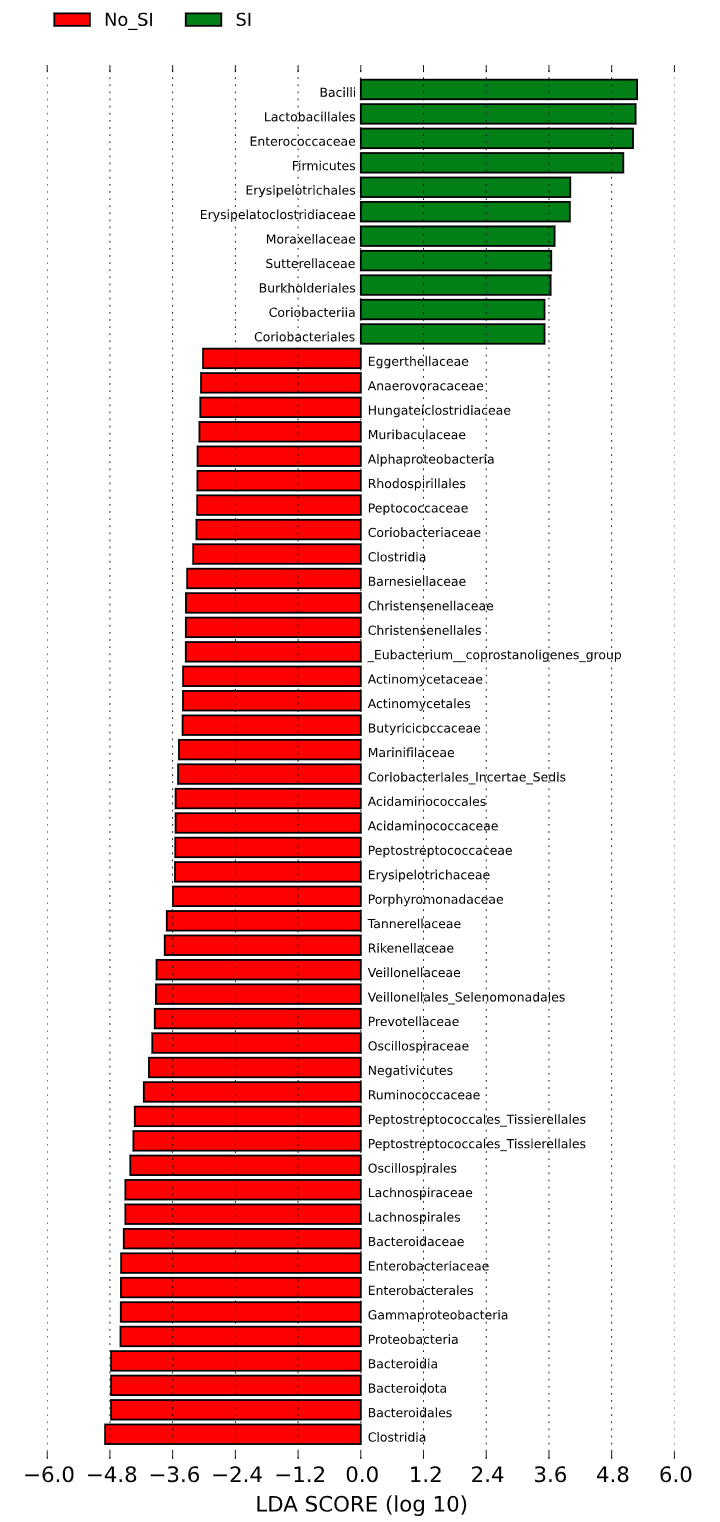


**Lower respiratory tract microbiome**

**Figure S3:** **Lower respiratory tract microbiome**. Plot of the top 15 relative bacterial abundances at genus level in the bronchoalveolar microbiome (BAL, left) and tracheal microbiome (ETA, right)

**Table S4a:** **Lower respiratory tract microbiome: relative abundance of the top taxa in the bronchoalveolar microbiome** **(BAL)**

| **Taxa** | **Full patient subset**^a^ | **Patients without SI**^b^ | **Patients with SI**^b^ | | ***q* value**^c^ |
| --- | --- | --- | --- | --- | --- |
|  |  |  | |  |  |
| **Class level** |  |  | |  |  |
| Bacilli | 41.91 (0.01 – 99.76) | 38.18 | 45.63 | | 0.8 |
| Gammaproteobacteria | 21.48 (0.01 – 99.65) | 26.82 | 16.15 | | 0.8 |
| Clostridia | 9.71 (0 – 47.52) | 8.63 | 10.79 | | 0.8 |
| Actinobacteria | 7.62 (0 – 53.48) | 9.56 | 5.69 | | 0.8 |
|  |  |  |  | |  |
| **Genus level** |  |  |  | |  |
| *Streptococcus* | 18.09 (0 – 98.67) | 18.03 | 18.15 | | 0.8 |
| *Enterococcus* | 10.13 (0 – 99.71) | 4.73 | 15.53 | | 0.8 |
| *Staphylococcus* | 3.85 (0 – 45.23) | 4.65 | 3.05 | | 0.9 |
|  |  |  |  | |  |

^a^ Mean (min – max) [%]

^b^ Mean [%]

^c^ Wilcoxon rank-sum test; false discovery rate correction for multiple testing

*SI* Secondary infection, *BAL* Bronchoalveolar lavage

**Table S4b:** **Lower respiratory tract microbiome: relative abundance of the top taxa in the tracheal microbiome** **(ETA)**

| **Taxa** | **Full patient subset**^a^ | **Patients without SI**^b^ | **Patients with SI**^b^ | | ***q* value**^c^ | |
| --- | --- | --- | --- | --- | --- | --- |
|  |  |  | |  | |  |
| **Class level** |  |  | |  | |  |
| Bacilli | 40.49 (0.33 – 99.94) | 43.33 | 37.65 | | | 0.8 |
| Gammaproteobacteria | 25.23 (0.02 – 98.93) | 29.99 | 20.46 | | | 0.5 |
| Clostridia | 9.64 (0 – 54.33) | 3.35 | 15.94 | | | 0.3 |
| Bacteroidia | 7.49 (0 – 38.95) | 5.55 | 9.43 | | | 0.5 |
| Actinobacteria | 6.93 (0 – 56.35) | 8.93 | 4.92 | | | 0.9 |
|  |  |  |  | | |  |
| **Genus level** |  |  |  | | |  |
| *Streptococcus* | 15.83 (0 – 99.28) | 24.81 | 6.9 | | | 0.1 |
| *Staphylococcus* | 6.28 (0 – 88.52) | 7.4 | 5.2 | | | 0.6 |
| *Enterococcus* | 9.58 (0 – 99.93) | 3.54 | 15.6 | | | 0.6 |
|  |  |  |  | | |  |

^a^ Mean (min – max) [%]

^b^ Mean [%]

^c^ Wilcoxon rank-sum test; false discovery rate correction for multiple testing

*SI* Secondary infection, *ETA* En­do­tracheal aspirate

**Table S4c:** **Lower respiratory tract microbiome: frequency of dominance (relative abundance ≥ 30%) among the most frequently dominant phyla in the lower respiratory tract microbiome**

| **Phylum level** | **n** | **Full patient subset**^a^ | **Patients without SI**^a^ | **Patients with SI**^a^ | ***q* value**^b^ |
| --- | --- | --- | --- | --- | --- |
|  |  |  |  |  |  |
| **Bronchoalveolar microbiome (BAL)** | | | | | |
|  | 54 |  |  |  |  |
| Actinobacteriota |  | 4 (7%) | 4 (10%) | 0 (0%) | > 0.9 |
| Firmicutes |  | 21 (39%) | 14 (36%) | 7 (47%) | > 0.9 |
| Proteobacteria |  | 6 (11%) | 6 (15%) | 0 (0%) | > 0.9 |
| Other |  | 1 (2%) | 1 (3%) | 0 (0%) | > 0.9 |
|  |  |  |  |  |  |
| **Tracheal microbiome (ETA)** | | | | | |
|  | 61 |  |  |  |  |
| Actinobacteriota |  | 3 (5%) | 3 (6%) | 0 | > 0.9 |
| Firmicutes |  | 31 (51%) | 26 (55%) | 5 (36%) | > 0.9 |
| Proteobacteria |  | 13 (21%) | 12 (26%) | 1 (7%) | > 0.9 |
| Other |  | 2 (3%) | 1 (2%) | 1 (7%) | > 0.9 |
|  |  |  |  |  |  |

^a^ n (%)

^b^ Fisher's exact test; Pearson's Chi-squared test; false discovery rate correction for multiple testing

*SI* Secondary infection, *BAL* Bronchoalveolar lavage, *ETA* En­do­tracheal aspirate

**Table S4d:** **Lower respiratory tract microbiome: α-diversity of the lower respiratory tract microbiome**

|  | **n** | **Full patient set**^a^ | **Patients without SI**^a^ | **Patients with SI**^a^ | ***p* value**^b^ |
| --- | --- | --- | --- | --- | --- |
|  |  |  |  |  |  |
| **Bronchoalveolar microbiome (BAL)** | | | | | |
| Shannon | 54 | 2.43 (1.64, 3.44) | 2.44 (1.74, 3.48) | 2.18 (1.33, 3.25) | 0.8 |
| Simpson | 54 | 0.83 (0.52, 0.95) | 0.82 (0.58, 0.95) | 0.88 (0.50, 0.95) | > 0.9 |
| ACE | 44 | 59 (44, 81) | 60 (53, 82) | 50 (42, 60) | 0.2 |
| Chao1^c^ | 54 | 58 (36, 79) | 60 (36, 80) | 48 (36, 62) | 0.2 |
| PD | 54 | 4.44 (3.41, 5.50) | 4.65 (3.56, 5.79) | 4.19 (2.74, 5.48) | 0.3 |
| SR^c^ | 54 | 58 (36, 79) | 60 (36, 80) | 48 (36, 62) | 0.2 |
|  |  |  |  |  |  |
| **Tracheal microbiome (ETA)** | | | | | |
| Shannon | 61 | 2.46 (1.58, 3.13) | 2.29 (1.49, 2.95) | 2.90 (1.71, 3.42) | 0.3 |
| Simpson | 61 | 0.83 (0.61, 0.93) | 0.83 (0.59, 0.92) | 0.93 (0.69, 0.96) | 0.1 |
| ACE | 47 | 53 (31, 104) | 54 (32, 105) | 49 (26, 74) | 0.4 |
| Chao1^c^ | 61 | 46 (27, 89) | 46 (29, 90) | 35 (22, 70) | 0.3 |
| PD | 61 | 3.49 (2.61, 6.21) | 3.49 (2.66, 5.87) | 3.57 (2.32, 6.02) | 0.8 |
| SR^c^ | 61 | 46 (27, 89) | 46 (29, 90) | 35 (22, 70) | 0.3 |
|  |  |  |  |  |  |

^a^ Median (IQR)

^b^ Wilcoxon rank-sum test; Wilcoxon rank-sum exact test

^c^ Chao1 and SR values were identical in BAL and ETA samples, respectively, due to the absence of singletons.

*SI* Secondary infection, *BAL* Bronchoalveolar lavage, *ETA* En­do­tracheal aspirate, *ACE* Abundance-based cover­age estimator, *PD* Phylogenetic Diversity, *SR* Species richness

**Figure S4a:** **Principal coordinate analysis (PCoA)** based on generalized UniFrac distances of the bronchoalveolar mi­crobiome (BAL) and tracheal microbiome (ETA). Solid ellipses indicate the 95% confidence intervals of the group centroids, while dashed ellipses represent the area expected to contain 95% of the data points for each group, assuming a multivariate normal distribution. The percentage of variance explained by each axis is shown.

**BAL**

Generalized UniFrac: R^2^ = 0.02, F = 0.83,

*p* = 0.69

Unweighted UniFrac: R^2^ = 0.02, F = 1.00,

*p* = 0.41

Weighted UniFrac: R^2^ = 0.01, F = 0.76,

*p* = 0.65

Bray-Curtis dissimilarity: R^2^ = 0.02, F = 1.07,

*p* = 0.31

**ETA**

Generalized UniFrac: R^2^ = 0.04, F = 2.15,

*p* < 0.01

Unweighted UniFrac: R^2^ = 0.06, F = 1.76,

*p* = 0.03

Weighted UniFrac: R^2^ = 0.04, F = 2.41,

*p* = 0.03

Bray-Curtis dissimilarity: R^2^ = 0.03, F = 1.85,

*p* < 0.01

**Figure S4b:** **Linear discriminant analysis (LDA) effect size (LEfSe) analysis of the bronchoalveolar microbiome (BAL).** The histogram shows the LDA score, with bar lengths indicating the magnitude of each LDA score, reflecting how strongly each taxon differentiates between groups.


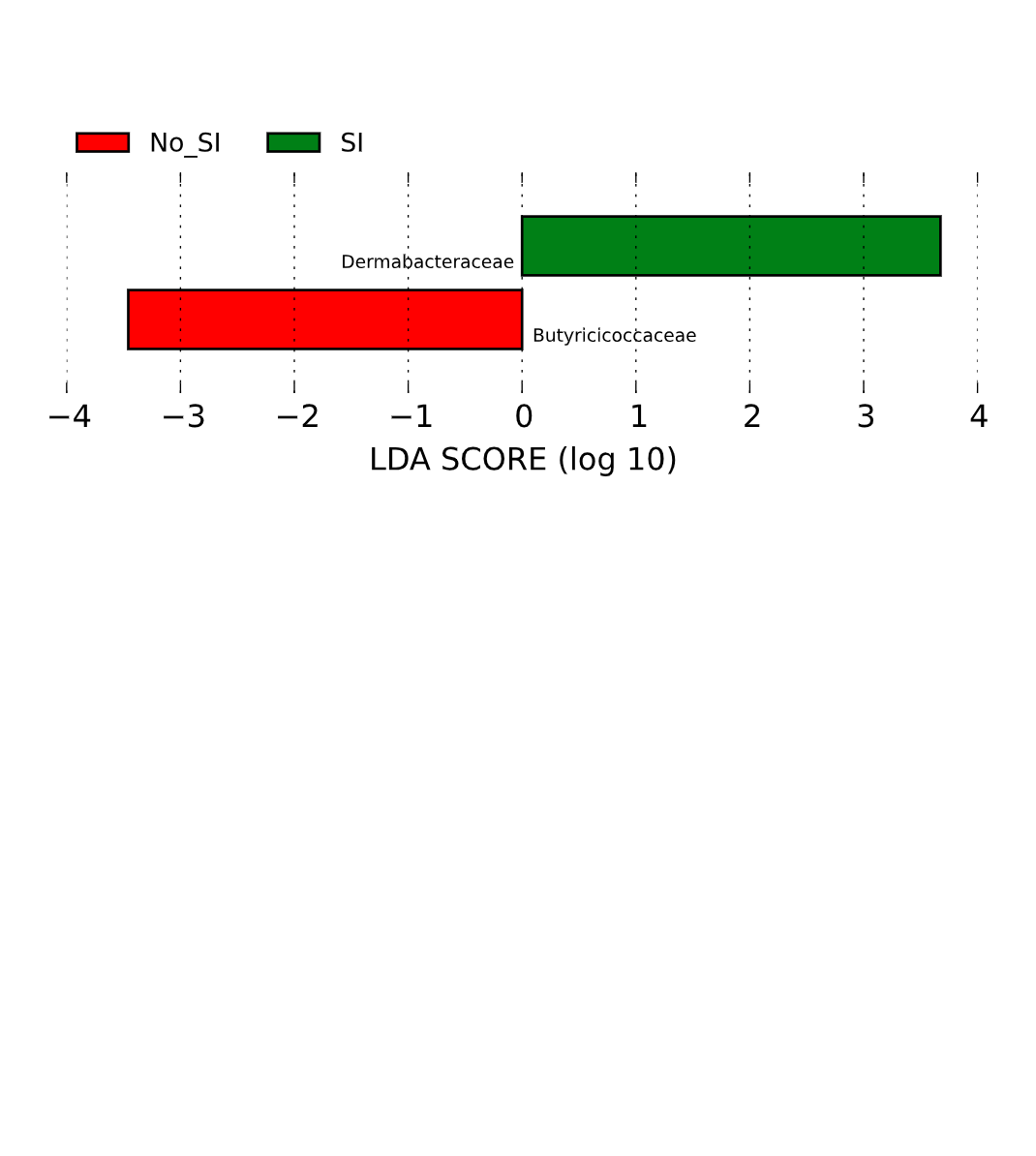


**Figure S4c: Linear discriminant analysis (LDA) effect size (LEfSe) analysis of the tracheal microbiome (ETA).** The cladogram illustrates the phylogenetic distribution of microbial taxa. The histogram shows the LDA score, with bar lengths indicating the magnitude of each LDA score, reflecting how strongly each taxon differentiates between groups.

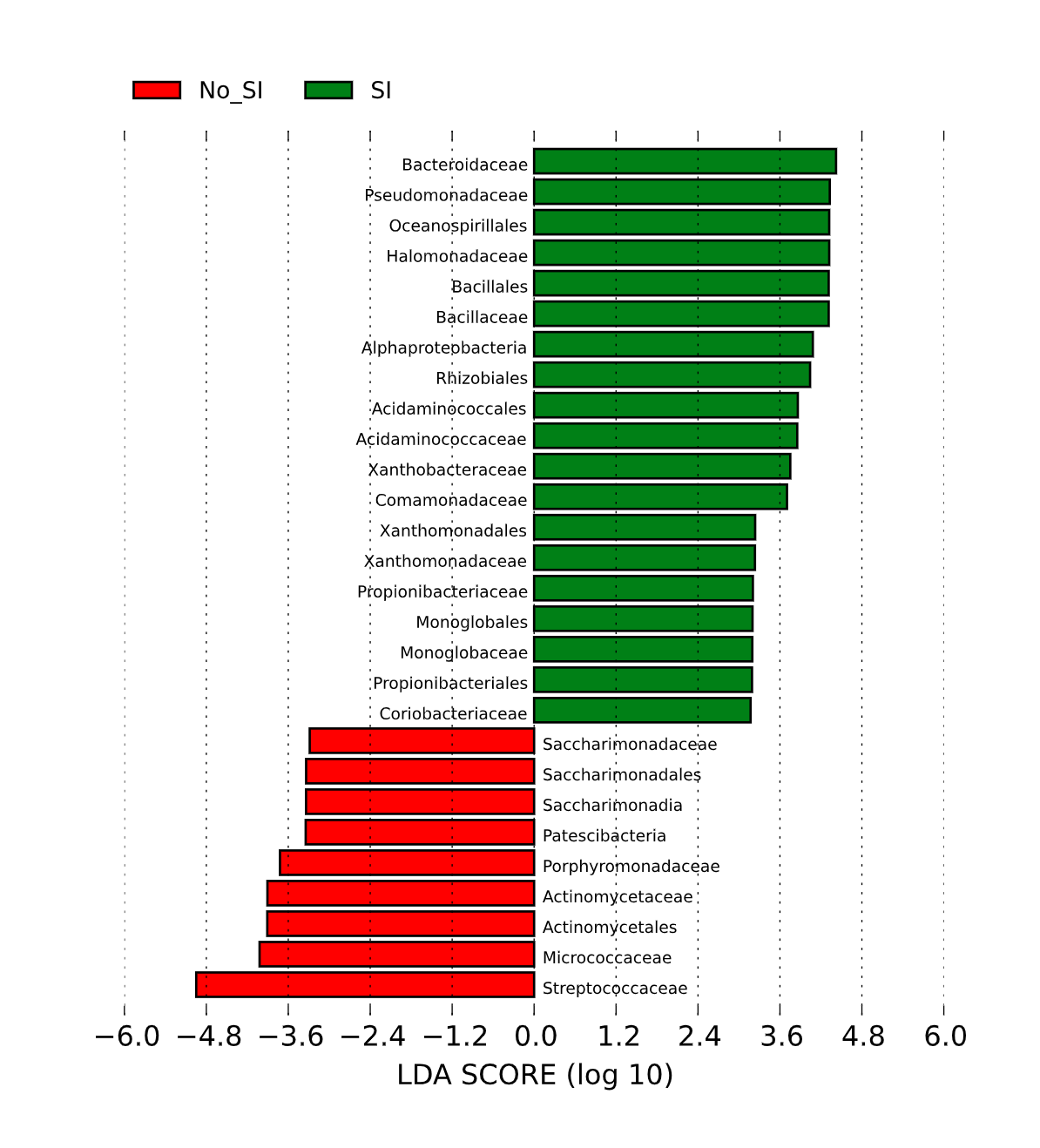


**References**

1. Kuo SZ, Dettmer K, Annavajhala MK, Chong DH, Uhlemann AC, Abrams JA, et al. Associations between urinary 3-indoxyl sulfate, a gut microbiome-derived biomarker, and patient outcomes after intensive care unit admission. J Crit Care. 2021;63:15-21.

2. Callahan BJ, McMurdie PJ, Rosen MJ, Han AW, Johnson AJA, Holmes SP. DADA2: High-resolution sample inference from Illumina amplicon data. Nat Methods. 2016;13(7):581-3.

3. Bolyen E, Rideout JR, Dillon MR, Bokulich NA, Abnet CC, Al-Ghalith GA, et al. Reproducible, interactive, scalable and extensible microbiome data science using QIIME 2. Nat Biotechnol. 2019;37(8):852-7.

4. McMurdie PJ, Holmes S. phyloseq: An R Package for Reproducible Interactive Analysis and Graphics of Microbiome Census Data. PLoS One. 2013;8(4):e61217.

5. Anderson MJ. A new method for non-parametric multivariate analysis of variance. Austral Ecol. 2001;26(1):32-46.
